# CD44+ Cancer Stem-Like Cells in EBV-Associated Nasopharyngeal Carcinoma

**DOI:** 10.1371/journal.pone.0052426

**Published:** 2012-12-21

**Authors:** Samantha Wei-Man Lun, Siu Tim Cheung, Phyllis Fung Yi Cheung, Ka-Fai To, John Kong-Sang Woo, Kwong-Wai Choy, Chit Chow, Chartia Ching-Mei Cheung, Grace Tin-Yun Chung, Alice Suk-Hang Cheng, Chun-Wai Ko, Sai-Wah Tsao, Pierre Busson, Margaret Heung-Ling Ng, Kwok-Wai Lo

**Affiliations:** 1 Department of Anatomical and Cellular Pathology, State Key Laboratory in Oncology in South China, Prince of Wales Hospital, The Chinese University of Hong Kong, Hong Kong SAR; 2 Li Ka Shing Institute of Health Science, The Chinese University of Hong Kong, Hong Kong SAR; 3 Department of Surgery, Li Ka Shing Faculty of Medicine, University of Hong Kong, Hong Kong SAR; 4 Department of Otorhinolaryngology, Head and Neck Surgery, The Chinese University of Hong Kong, Hong Kong SAR; 5 Department of Obstetrics and Gynaecology, Prince of Wales Hospital, The Chinese University of Hong Kong, Hong Kong SAR; 6 Department of Anatomy, Li Ka Shing Faculty of Medicine, University of Hong Kong, Hong Kong SAR; 7 Université Paris-Sud-11, CNRS-UMR 8126 and Institut de cancérologie Gustave Roussy, Villejuif, France; Johns Hopkins University, United States of America

## Abstract

Nasopharyngeal carcinoma (NPC) is a unique EBV-associated epithelial malignancy, showing highly invasive and metastatic phenotype. Despite increasing evidence demonstrating the critical role of cancer stem-like cells (CSCs) in the maintenance and progression of tumors in a variety of malignancies, the existence and properties of CSC in EBV-associated NPC are largely unknown. Our study aims to elucidate the presence and role of CSCs in the pathogenesis of this malignant disease. Sphere-forming cells were isolated from an EBV-positive NPC cell line C666-1 and its tumor-initiating properties were confirmed by *in vitro* and *in vivo* assays. In these spheroids, up-regulation of multiple stem cell markers were found. By flow cytometry, we demonstrated that both CD44 and SOX2 were overexpressed in a majority of sphere-forming C666-1 cells. The CD44+SOX2+ cells was detected in a minor population in EBV-positive xenografts and primary tumors and considered as potential CSC in NPC. Notably, the isolated CD44+ NPC cells were resistant to chemotherapeutic agents and with higher spheroid formation efficiency, showing CSC properties. On the other hand, microarray analysis has revealed a number of differentially expressed genes involved in transcription regulation (e.g. *FOXN4*, *GLI1*), immune response (*CCR7*, *IL8*) and transmembrane transport (e.g. *ABCC3*, *ABCC11*) in the spheroids. Among these genes, increased expression of CCR7 in CD44+ CSCs was confirmed in NPC xenografts and primary tumors. Importantly, blocking of CCR7 abolished the sphere-forming ability of C666-1 *in vitro*. Expression of CCR7 was associated with recurrent disease and distant metastasis. The current study defined the specific properties of a CSC subpopulation in EBV-associated NPC. Our findings provided new insights into developing effective therapies targeting on CSCs, thereby potentiating treatment efficacy for NPC patients.

## Introduction

Non-keratinizing nasopharyngeal carcinoma (NPC) is a distinct epithelial malignancy arising from the head and neck region. It consistently associates with Epstein-Barr virus (EBV) and shows unique clinical and pathological feature. With the clonal expansion of a single EBV-infected progenitor cell, constitutive expression of viral latent genes and accumulation of multiple genetic changes contribute to the initiation and progression of this cancer [1], [2].

NPC demonstrates strong geographic preference, showing high prevalence in Southeast Asia, especially in the Cantonese region including Hong Kong with an annual incidence of 25–50 per 100,000 persons [1]. The mainstay treatment for NPC is either radiotherapy or combined chemo-radiotherapy which shows over 90% cure rate in patients with early disease stage [3]. However, the outcome for patients with advanced loco-regional diseases and distant metastases are unsatisfactory. Significant rates of distant relapse and metastasis still occur in these patients after radiotherapy or chemo-radiotherapy. One of the major mechanisms for such post-therapeutic recurrence of NPC has been suggested by the ‘cancer stem-like cell’ (CSC) proposition [4].

According to the CSC model, cancers are hierarchically organized similar to normal tissues and cancer growth and progression are driven by a small subset of tumor cells with stem cell-like properties, the CSCs. This rare cell subpopulation is responsible for tumor initiation, maintenance and regeneration [4], [5]. CSCs have been identified in various human malignancies such as breast, prostate, ovarian, lung and head and neck carcinoma [5], [6], [7], [8], [9]. The abilities of these cells to initiate tumor growth, sustain self-renewal and facilitate drug resistance have been extensively proven. Considering the unique properties of CSCs, the relapse of tumors are suggested to be due to the failure to eradicate all CSCs and surviving CSCs then reconstitute the tumor in local and distant regions.

Although CSCs have been shown to be vital in the development of most cancers, information regarding their existence in EBV-associated NPC is scarce. Either research was conducted using EBV-negative cell lines or was unable to elucidate any functional CSCs [10], [11], [12]. Our group has previously established several EBV-positive tumor lines from NPC patients in endemic regions [13], [14], [15], [16] and using these native EBV-positive tumor cells, we aimed to identify and characterize functional NPC CSCs.

In this study, sphere-forming cells (‘spheroids’) from EBV-positive cell line C666-1 were isolated and their stem cell-like properties were confirmed. We revealed that CD44 and SOX2 were expressed in a majority of these CSCs. By microarray analysis, aberrantly expressed cellular genes in CSCs were identified. Interestingly, CCR7, a cell surface chemokine receptor, was found to be consistently expressed in NPC lines and primary tumors. Coincidentally, it was overexpressed in our identified NPC CSCs and the neutralization of this receptor abolished the sphere-forming ability of C666-1. Our current study provided first evidence for the presence of tumorigenic CSCs in EBV-positive NPC and CCR7 might play a role in their tumorigenic function. The above findings will enhance our understanding on the nature of NPC CSCs, which is crucial in developing more effective therapeutic intervention against this disease.

## Results

### CSC Capabilities of EBV-positive NPC-derived Spheroids

**Figure 1:**
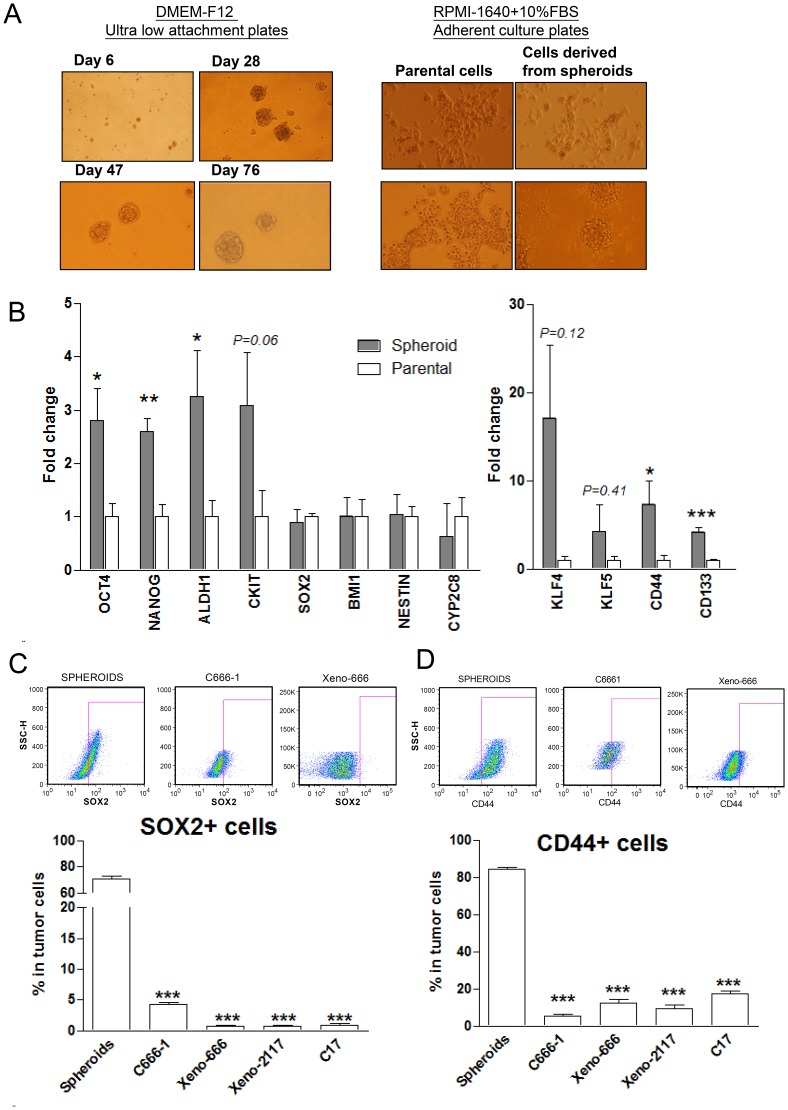
Sphere-forming cells with stem cell-like properties in EBV-positive NPC. (A) Free-floating tumor spheres were formed from EBV-positive C666-1 cells (left panel) and they demonstrated ability to differentiate comparably to monolayer cells upon administering complete medium (right panel). (B) By qRT-PCR, multiple stem cell-related genes (OCT4, NANOG, ALDH1, CKIT, CD44, CD133) were enriched in spheroids when compared to parental C666-1. Transcription of SOX2 was not increased in the spheroids. (C) SOX2 protein was frequently expressed in C666-1 sphere-forming cells. By flow cytometry, SOX2-positive (SOX2+) cells were found to be enriched in and constituted over 60% of sphere-forming cell population. (D) Over 80% of sphere-forming cells expressed cell surface marker CD44 while CD44+ cells in detected in parental C666-1 and other NPC xenografts were significantly lower (all *P*<0.001). Histograms denoting mean ± SE (n≥3) with statistical significance calculated by t-test (*P<0.05, **P<0.01, ***P<0.001).

CSCs possessed the capacity to form anchorage-independent tumor spheres when grown in specialized serum-free medium. As shown in Figure 1A, free-floating tumor spheres were formed when the dissociated C666-1 cells were cultured on uncoated plates in serum-free stem cell medium. Non-adherent spheroids were observable at 6–8 days and they were enzymatically dissociated into single cells for passage weekly. Distinct prototypical spheroids were formed 28 days after plating. This self-renewing floating tumor spheres could be passaged serially and grown for more than 3 months. When single cells from spheroids was cultured in adherent plates with complete medium (RPMI-1640 with 10% FBS), floating cells adhered to the plate and formed systematic colonies, showing epithelial cell morphology similar to that of parental monolayer C666-1 cells (Fig. 1A, right panel).

To measure the tumorigenic capacity of spheroids, various numbers of sphere-forming cells and unselected parental C666-1 cells were injected subcutaneously into flanks of nude mice. We found that 10,000 sphere-forming cells could form tumors in all inoculated mice (Table 1). Injection of 1,000 spheroid cells occasionally formed one tumor out of six mice. However, at least 500,000 unselected C666-1 cells were necessary for tumor formation. The spheroid cells showed at least 50 times higher tumorigenic potential than the unselected cells. To further verify whether the sphere-forming cells are capable of serially propagating in nude mice, xenografts developed from spheroids were serially engrafted into nude mice. All of them showed successful serial transplantations and tumors were observable at 3–4 weeks (data not shown).

**Table 1:** In vivo tumorigenic capacity of sphere-forming cells and unselected parental cells of C666-1 in nude mice.

Sample type	No. of cells/injection	No. of tumor formed/injection	Latency (days)	Sample type	No. of cells/injection	No. of tumor formed/injection	Latency (days)
Sphere-forming cells	100	0/6	N/A	Parental C666-1	10,000	0/6	N/A
	500	0/6	N/A		100,000	0/6	N/A
	1,000	1/6	54		500,000	5/6	28
	5,000	3/6	69		1,000,000	4/6	28
	10,000	4/4	39		5,000,000	6/6	14

N/A – data not available.

We then examined the expression pattern of multiple stem cell markers and surface antigens in these spheroids. By qRT-PCR analysis, we found that the expression of multiple stem cell markers (OCT4, NANOG, ALDH1, CD44 and CD133) in spheroids was significantly higher than that of the monolayer parental C666-1 cells (all *P*<0.05, Fig. 1B). Increased expression of CKIT, KLF4 and KLF5 in the spheroids was also noted. Although we did not detect up-regulation of SOX2 transcription in the sphere-forming cells, SOX2-expressing (SOX2+) cells were shown to be highly enriched in the spheroids (70.8±2.16%) by flow cytometry (all *P*<0.001, Fig. 1C). We also detected SOX2 expression in 0.77±0.13% of cells in NPC xenografts. The findings confirmed that the spheroids derived from EBV-positive C666-1 cells exhibit CSC phenotypes.

### Sphere-forming CSCs Express Cell Surface Protein CD44

Since the transcription of CD44 and CD133 were significantly increased in spheroids, we then verified whether the expression of these CSCs surface markers was specific to NPC sphere-forming cells. By flow cytometry and immunofluorescence staining, we have demonstrated that a majority of the cells in C666-1 spheroids (84.14±1.12%) were CD44 positive (Fig. 1D and Fig. S1). CD44+ cells were also found as a subpopulation of the parental C666-1 cells (5.28±1.29%) and three NPC xenografts, xeno-666 (12.31±1.97%), xeno-2117 (9.58±1.80%), and C17 (17.31±1.76%) (Fig. 1D). However, CD133-expressing cells were detected in only 32.11±2.35% of spheroids cells and 1.90±0.84% of parental C666-1 cells. Furthermore, CD133+ cells were completely absent in 2 of the xenografts (xeno-666 and xeno-2117) (Fig. S2). Thus, we proposed that CD44 is a common candidate CSC surface marker for EBV-positive NPC.

### CD44+ Cells Express SOX2 and Exhibit Higher Clone- and Sphere-forming Efficiency

**Figure 2:**
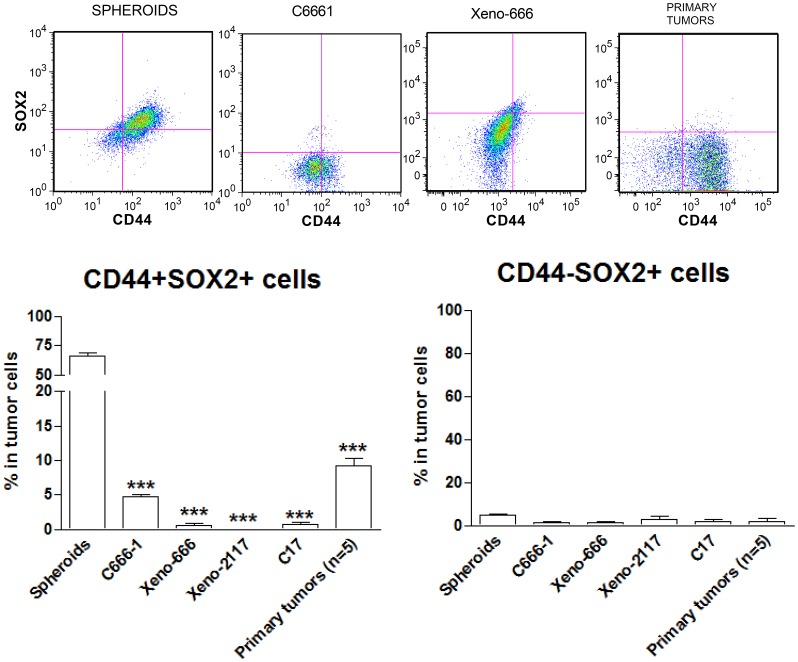
CD44 and SOX2 as CSC markers. By flow cytometry, SOX2 was found to be preferentially expressed on CD44+ cells and coincidentally, SOX2 expression was rarely detected in CD44− cells. Cells coexpressing both CD44 and SOX2 were found to be enriched in spheroids. Histograms denoting mean ± SE (n≥3) with statistical significance calculated by t-test (*P<0.05, **P<0.01, ***P<0.001).

**Figure 3:**
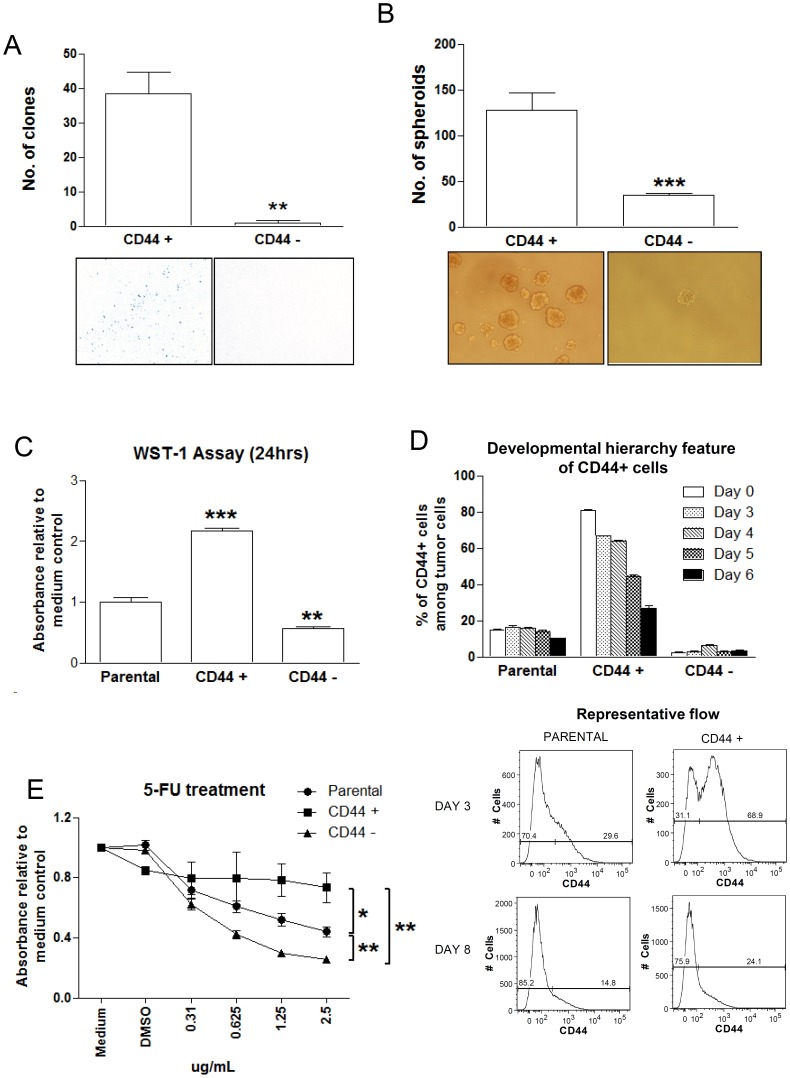
CD44+ cells with stem cell-like properties in EBV-positive NPC. CD44+ cell fraction exhibited a significantly higher (A) clone formation efficiency and (B) sphere-forming efficiency when compared to CD44− cell fraction. In addition, (C) CD44+ cells exhibited significantly higher proliferation rate than CD44− cells. (D) Developmental hierarchy feature of CD44+ cells. Percentage of CD44+ cells were continually reduced in the isolated CD44+ cell fraction over time. (E) CD44+ cells exhibited higher resistance to 5-FU treatment when compared to the CD44− and parental C666-1 cells. All graphs denoting mean ± SE (n≥3) with statistical significance calculated by t-test (*P<0.05, **P<0.01, ***P<0.001).

Expression of stem cell transcription factor SOX2 was consistently detected in a tumor cell sub-population in primary NPC and is believed to be a potential marker for NPC CSCs [12]. Consequently, we assessed the co-expression of CD44 and SOX2 in spheroids and unselected monolayer C666-1 cells, as well as 3 xenografts and 5 primary tumors by flow cytometry. As shown in Figure 2, over 60% of spheroid cells (66.57±2.72%) expressed both CD44 and SOX2. Coincidentally, SOX2 expression was rarely detected in CD44− fraction of C666-1 sphere-forming cells, suggesting a preferential expression of this stem cell transcription factor in CD44+ cells. Co-expression of CD44 and SOX2 was also detected in 0.1–9% of cells in the NPC cell line, xenografts and primary tumors. The findings implied that CD44+SOX2+ cells are potential CSCs in NPC. To prove this hypothesis, we performed functional assays in CD44+ and CD44− cells isolated from parental C666-1 cells. Representative figures of CD44 expression in the isolated CD44+ and CD44− cells are shown in Figure S3. As shown in Figure 3A, significantly higher clone formation efficiency was detected in the CD44+ cell fraction than that of CD44− fraction (*P*<0.01). Moreover, CD44+ cells could generate significantly higher number of spheroids compared with the CD44− cells (*P*<0.001, Fig. 3B).

### Proliferation of CD44+ Cells

By WST1 proliferation assay, CD44+ cells exhibited a significantly higher proliferation rate when compared to the CD44− and parental C666-1 cells, while CD44− cells showed a significantly lower proliferation rate when compared to parental C666-1 cells (all P<0.01, Fig. 3C).

### Hierarchical Relationship of CD44+ and CD44− Cells

Consistent with CSC properties, we revealed that the sorted CD44+ C666-1 cells possessed the ability to give rise to both CD44+ and CD44− cells. As shown in Fig. 3D, a decrease in CD44+ cell fraction in the sorted CD44+ cells was detected by flow cytometry over time. It is likely that sorted CD44+ cells give rise to a heterogeneous population through downregulation of CD44 in a subset of these cells after culture *in vitro.* However, no significant change in the percentage of CD44+ cell fraction in both unsorted parental and sorted CD44− cells was detected after 3–6 days of passage. The results suggest a hierarchical relationship of CD44+ and CD44− cells in EBV-associated NPC.

### Chemoresistance of CD44+ Cells

To examine the chemoresistance of CD44+ cells to anti-cancer chemotherapeutic agents, we performed cell survival assays for the CD44+ and CD44− cell fractions treated with fluorouracil (5-FU), cisplatin or doxorubicin. The CD44+ cell fraction of C666-1 exhibited stronger chemoresistance to 5-FU compared with the parental unselected C666-1 cells and CD44− fraction (all *P*<0.05) (Fig. 3E). The chemoresistance of CD44+ cells towards cisplatin or doxorubicin was similar to CD44− and parental C666-1 cells (data not shown).

### Differentially Expressed Genes in NPC-derived Spheroids

**Figure 4:**
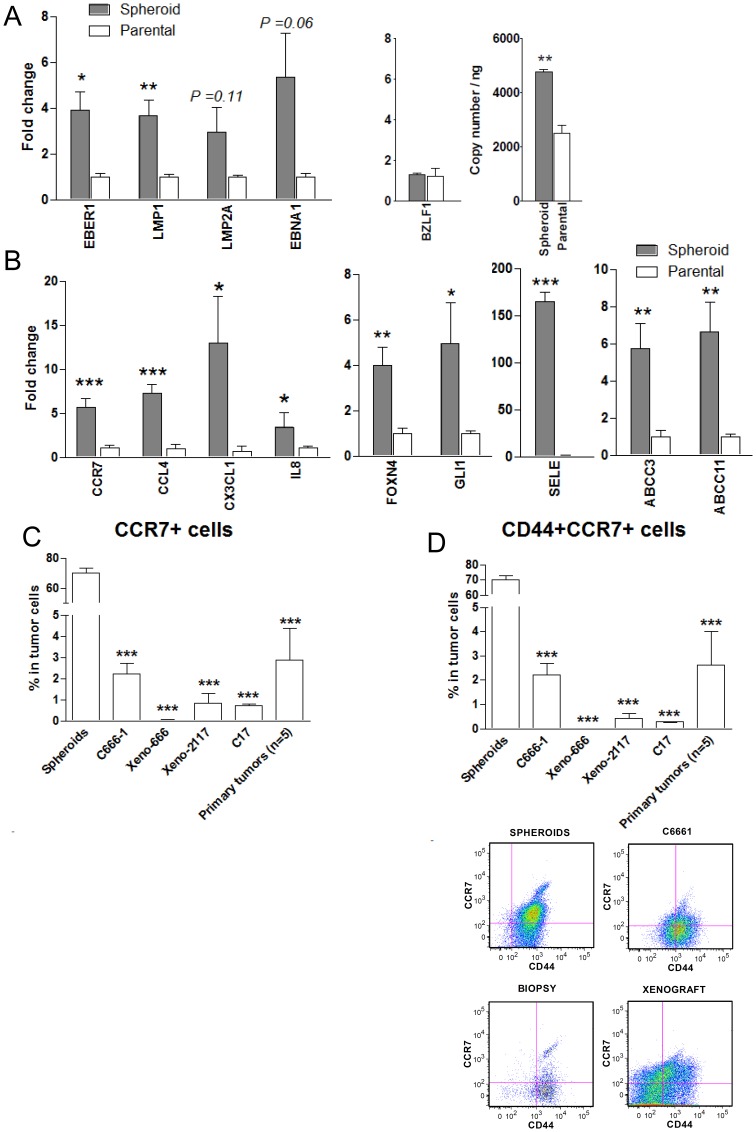
Overexpression of multiple EBV and cellular genes in sphere-forming NPC cells. (A) By qRT-PCR, multiple EBV genes (EBER, BARF1, LMP1, LMP2A, EBNA1 and BZLF1) were found to be overexpressed in spheroids when compared to monolayer C666-1 cells. EBV copy number in these cells was determined by qPCR. (B) Selected genes aberrantly expressed in spheroids were confirmed by qRT-PCR. The significantly upregulated genes include chemokines and receptors (CCR7, CCL4, CX3CL1 and IL-8), cell adhesion molecule SELE, signaling molecules (GLI1, FOXN4) and ABC transporters (ABCC3, ABCC11). (C) Cell surface-expressed CCR7 was found to be frequently expressed in sphere-forming cells (>60%) by flow cytometry. The CCR7+ cell subpopulation was also detected in NPC lines and primary tumors (<5%). (D) CD44+CCR7+cells were also found to be enriched in spheroids. Histograms denoting mean ± SE (n≥3) with statistical significance calculated by t-test (*P<0.05, **P<0.01, *** *P*<0.001).

To comprehensively characterize the properties of NPC CSCs, we examined the expression of cellular and EBV genes in NPC sphere-forming cells using olignonucleotide microarray and qPCR assays. Firstly, we assessed EBV copy number and the expression level of EBV genes including EBNA1, LMP1, LMP2A, BARF1, EBER1 and BZLF1 in C666-1 spheroids. The sphere-forming cells showed higher EBV copy number and latent gene expression than the parental C666-1 cells (Fig. 4A). We detected significant elevation of EBER1, BARF1 and LMP1 expression in the spheroids (all *P*<0.05). No significant difference in the expression of EBV immediate-early lytic gene BZLF1 was observed between the spheroids and parental C666-1 cells.

The cellular genes differentially expressed in spheroids and monolayer C666-1 cells were identified by microarray analysis. Expression of 830 and 260 genes were found to show more than 5-fold increase and decrease in the sphere-forming cells respectively. Gene ontology analysis revealed that the 5 major molecular function catalogs enriched in the differentially expressed genes include: regulation of transcription, immune response, regulation of apoptosis, cell adhesion, and transmembrane transport, each with significant P values (Table 2 and Table S2). A selection of differentially expressed genes was listed in Table 2. By qRT-PCR, we validated the expression of selected genes involved in maintaining stem cell properties in the spheroids and parental C666-1 (Fig. 4B). Significantly increased expression (*P*<0.05) of chemokines and receptors (IL8, CCL4, CX3CL1, CCR7), signaling pathway-related genes (FOXN4 and GLI1), and cell adhesion molecules (SELE) were confirmed in the spheroids (Fig. 4B). Notably, two ATP-binding cassette (ABC) transporter genes (ABCC3 and ABCC11) are also highly expressed in the spheroids (all *P*<0.01). The overexpression of these genes could be responsible for the chemoresistant feature of NPC CSCs (Fig. 4B).

**Table 2:** Selection of aberrantly expressed genes in sphere-forming cells compared to monolayer C666-1 cells.

Genes	Gene Description	Genbank Accession no.	Fold Changes
**Regulation Transcription (P value: 0.0041; n = 132)**			
FOXN4	Forkhead box N4	NM_213596	43.28
EGR1	Early growth response 1	NM_001964	33.48
HOXA7	Homeobox A7	NM_006896	20.43
STAT4	Signal transducer and activator of transcription 4	NM_003151	11.99
NFATC1	Nuclear factor of activated T-cells, cytoplasmic, calcineurin-dependent 1 (NFATC1), transcript variant 3	NM_172387	8.97
GLI1	Glioma-associated oncogene homolog 1 (zinc finger protein)	NM_005269	8.84
PAX6	Paired box gene 6 (aniridia, keratitis), transcript variant 2	NM_001604	7.70
RUNX1	Runt-related transcription factor 1 (acute myeloid leukemia 1; aml1 oncogene), transcript variant 2	NM_001001890	5.25
MBD2	Methyl-CpG binding domain protein 2, transcript variant testis-specific	NM_015832	−22.31
**Immune response (P value: 0.0051; n = 54)**			
CCR7	Chemokine (C-C motif) receptor 7 (CCR7)	NM_001838	169.42
IL1B	Interleukin 1, beta	NM_000576	43.24
CCL4	Chemokine (C-C motif) ligand 4 (CCL4)	NM_002984	20.85
RAG1	Recombination activating gene 1	NM_000448	16.95
IL7R	Interleukin 7 receptor	NM_002185	13.75
CX3CL1	Chemokine (C-X3-C motif) ligand 1 (CX3CL1)	NM_002996	9.49
IL8	Interleukin 8	NM_000584	8.00
CD86	CD86 molecule, transcript variant 2	NM_006889	7.78
TLR7	Toll-like receptor 7	NM_016562	5.28
HLA-G	HLA-G histocompatibility antigen, class I, G	NM_002127	−6.94
**Cell adhesion (P value: 0.032; n = 50)**			
SELE	Selectin E	NM_000450	149.51
TNC	Tenascin C	NM_002160	14.62
VCAN	Versican	NM_004385	8.42
ADAM12	ADAM metallopeptidase domain 12 (meltrin alpha), transcript variant 1	NM_003474	7.41
SLAMF7	SLAM family member 7	NM_021181	6.47
ICAM5	intercellular adhesion molecule 5, telencephalin	NM_003259	−10.99
RHOB	ras homolog gene family, member B	NM_004040	−10.80
**Transmembrane Transport (P value: 0.0041; n = 37)**			
SLC22A15	Solute carrier family 22 (organic cation transporter), member 15	NM_018420	27.91
SLC24A3	Solute carrier family 24 (sodium/potassium/calcium exchanger), member 3	NM_020689	24.87
SLC22A4	Solute carrier family 22 (organic cation transporter), member 4	NM_003059	14.45
ABCC11	ATP-binding cassette, sub-family C (CFTR/MRP), member 11, transcript variant 2	NM_033151	11.99
SLC16A6	Solute carrier family 16, member 6 (monocarboxylic acid transporter 7)	NM_004694	8.98
PDPN	Podoplanin (PDPN), transcript variant 1,	NM_006474	7.58
ABCC3	ATP-binding cassette, sub-family C (CFTR/MRP), member 3		5.34
**Positive regulation of apoptosis (P value: 0.026; n = 34)**			
MAL	Mal, T-cell differentiation protein (MAL), transcript variant a	NM_002371	17.84
BCL11B	B-cell CLL/lymphoma 11B (zinc finger protein), transcript variant 1	NM_138576	10.52
CASP1	Caspase 1, apoptosis-related cysteine peptidase (interleukin 1, beta, convertase), transcript variant alpha	NM_033292	9.30
HDAC6	Histone deacetylase 6	BC011498	5.70
CUL3	Cullin 3	NM_003590	5.29
CASP8	Caspase 8, apoptosis-related cysteine peptidase, transcript variant E	NM_033358	5.08
TNFRSF10B	Tumor necrosis factor receptor superfamily, member 10b	NM_003842	−5.81
TIA1	TIA1 cytotoxic granule-associated RNA binding protein, transcript variant 1	NM_022037	−6.59

### Expression of CCR7 in CD44+ CSCs of NPC

Among the differentially expressed genes, CCR7, a chemokine receptor, showed the highest fold change in expression in the spheroids by microarray analysis and was thus selected for further investigation. By flow cytometry, a significant enrichment of CCR7+ cells was detected in spheroids when compared to parental cells (all *P*<0.001, Fig. 4C). We also detected CCR7 expression in 0.53±0.14% and 2.87±1.53% of cells in xenografts and primary tumors respectively. Multicolor flow cytometric analysis revealed that a majority of CCR7+ cells were co-expressed with CD44. CD44+CCR7+ cells were enriched in spheroids when compared to the xenografts and primary tumors (all *P*<0.001, Fig. 4D). This candidate CSC fraction was also detected in NPC tumor lines and primary tumors.

**Figure 5:**
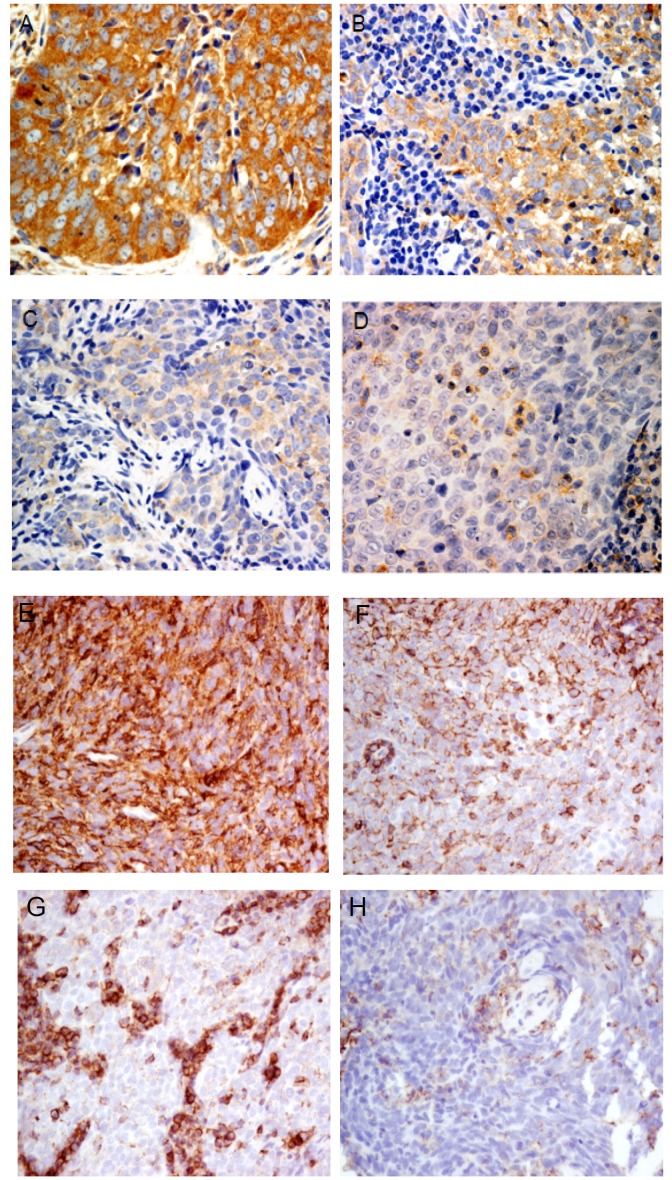
Immunohistochemical analysis of CCR7 and CD44 expression in primary NPC. Representative primary NPC cases with high (A), medium (B), low (C) expression of CCR7. (D) Primary NPC with absence of CCR7 expression was shown. CCR7 staining were detected in few infiltrating lymphocytes, but not in the tumor cells. Primary tumors with high (E) and medium (F) CD44 expression were shown. In (G) and (H), weak CD44 expression was detected in the tumor cells while strong CD44 staining in infiltrating lymphocytes was commonly found.

Since CCR7 was highly expressed in NPC sphere-forming cells and correlated with lymph node metastasis in various human cancers, we then determined the association of CCR7 expression with the clinical parameters in 39 primary NPC cases. Representative CCR7 expression patterns in primary tumors were shown in Figure 5. CCR7 expression is significantly correlated with CD44 expression in these cases (Fig. S4). Among the NPC samples, 8 (20.5%) were negative for CCR7 staining. Weak, medium and high expression of CCR7 were detected in 13, 7 and 11 primary cases respectively. Importantly, we found that CCR7 expression correlated with the presence of recurrent disease and distant metastasis (Table 3). The finding implied that the CCR7-expressing cells might contribute to disease progression.

**Table 3:** Correlation between CCR7 expression and clinicopathological features in primary tumors.

Variables	No. of patients	CCR7 expression score(mean, {no. of patients})		*P*-value
		Score = 0 group	Scores >0 group	
**Age (years)**				
≤50	20	0 {2}	4.7 {18}	*P* = 0.99
>50	19	0 {6}	5.7 {13}	
**Gender**				
Male	30	0 {7}	4.9 {23}	*P* = 0.05
Female	9	0 {1}	5.9 {8}	
**Clinical stage**				
Early (Stage 1,2)	14	0 {3}	6.5 {11}	*P* = 0.32
Late (Stage 3,4)	25	0 {5}	4.4 {20}	
**Recurrence/distant metastasis**				
Absent	30	0 {7}	5.5 {23}	* *P* = 0.03
Present	9	0 {1}	4.1 {8}	

N/A: Data not available.

* Spearman correlation test applied, with P-value <0.05 considered statistical significant.

**Figure 6:**
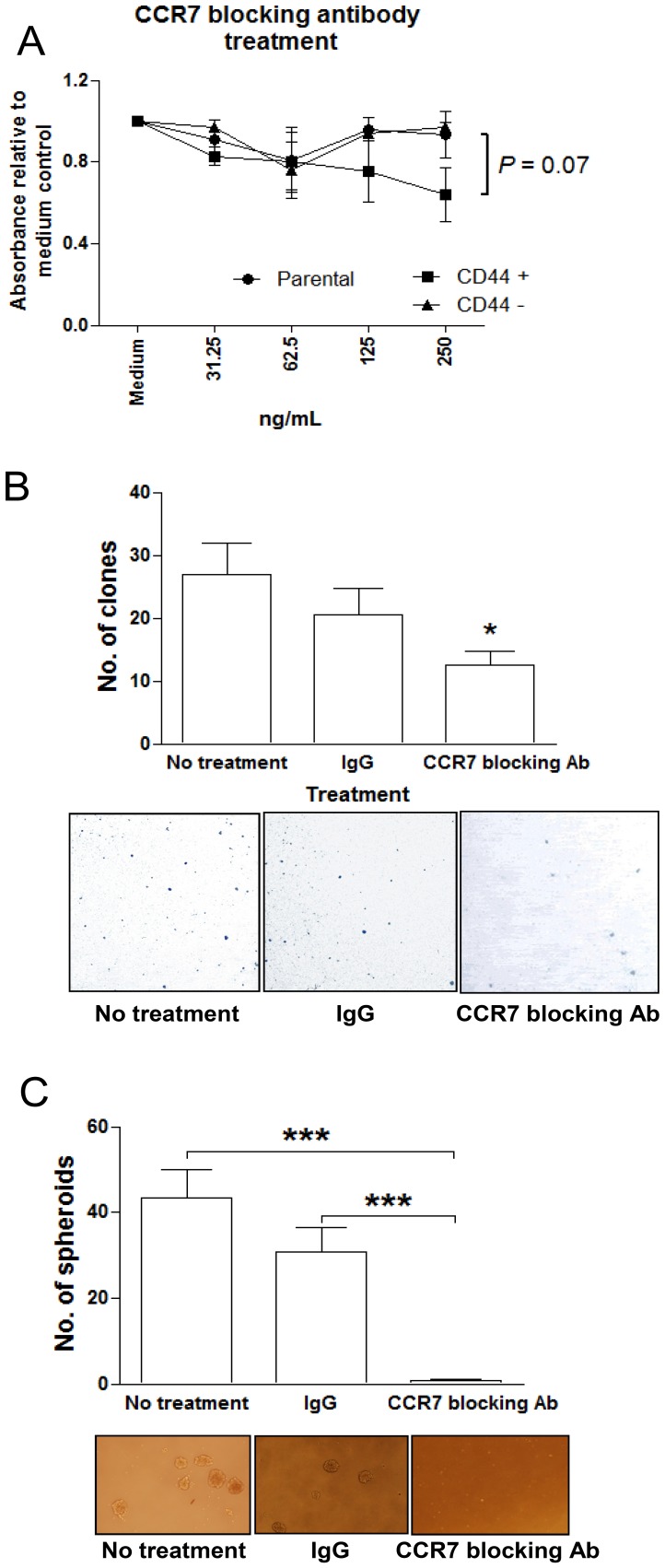
Effect of CCR7 neutralization on NPC CSCs. To evaluate the function of CCR7 in CSCs, C666-1 was treated with CCR7 blocking antibody and its proliferation, clone-forming and sphere-forming efficiency were investigated. (A) Proliferation of CD44+ cells was inhibited after treatment with CCR7 blocking antibody. (B) The clone formation efficiency of C666-1 cells was diminished after CCR7 blocking and (C) the spheroid-forming ability was significantly inihibited (*P*<0.001) when compared to untreated controls. Histograms denoting mean ± SE (n≥3) with statistical significance calculated by t-test (*P<0.05, ***P<0.001).

To investigate the role of CCR7 in NPC CSC, we utilized a CCR7-blocking antibody to neutralize its function in the parental, CD44+ and CD44− C666-1 cells. The cells were treated with 0–250 ng/mL CCR7 blocking antibody for 24 hrs and the proliferation of cells were measured by WST1 assay. Observable decrease in cell proliferation was observed in the CD44+ cell fraction when compared to CD44− and parental C666-1 cells. (Fig. 6A). Reduced clone formation efficiency was also found in C666-1 cells treated with CCR7 blocking antibody (*P*<0.05, Fig. 6B). Importantly, sphere-forming capability was significantly inhibited after CCR7 blocking antibody treatment (*P*<0.001, Fig. 6C). The results provided evidences for the involvement of CCR7 in maintaining CSC function in NPC.

## Discussion

This study provides the first evidence on the existence of highly tumorigenic CSCs in EBV-positive NPC. We have developed a protocol to obtain EBV-positive NPC CSCs for further characterization. Formation of spheroids is a major functional characteristic of CSCs in human cancers [17]. In this study, we successfully isolated a potential CSC population in the EBV-positive NPC cell line C666-1 through harvesting tumor spheres. The stem cell-like properties in sphere-forming C666-1 subpopulation obtained were confirmed by demonstrating the up-regulation of multiple stem cell-associated genes and high tumorigenicity in nude mice [9], [18], [19]. Through comprehensive characterization of the NPC spheroids, tumor lines and primary tumor, we have revealed that CD44 and SOX2 are potential CSC markers for NPC. Cell surface markers are crucial in identifying CSCs from among the heterogenic tumor cells. CD44 is a hyaluronan receptor and has been demonstrated to be marker for CSCs in head and neck squamous cell carcinoma (HNSCC) [7]. In this study, we showed that CD44 expression was enriched in NPC CSCs. As evidenced from the significantly higher sphere-forming capability of CD44+ NPC cells, CSC subpopulation was confirmed to be enriched in this CD44+ fraction. The resistance of CD44+ cells to 5-FU treatment is in concordance to proposed CSC properties. In addition to CD44, our study also detected the enrichment of SOX2-expressing cells in the spheroids, the functional NPC CSCs. Interestingly, similar level of SOX2 transcripts was found in both sphere-forming and parental C666-1 cells. According to our unpublished finding, the increased SOX2 protein expression may be due to the loss of the SOX2-repressing miRNAs (miR-183 and miR-203) in the spheroids. SOX2 is a transcription factor that controls pluripotency in embryonic and adult-tissue specific stem cells [20]. It regulates stem cell self-renewal and differentiation and is frequently found to be up-regulated in human cancers [21]. Zhang *et al*. (2010) has suggested that SOX2+ cells possess stemness properties [12]. They have demonstrated that SOX2 was expressed and co-localized with OCT4 in primary NPC. In concordance with Zhang’s findings, our results revealed that CD44+ SOX2+ cells are enriched in NPC CSCs.

Isolation of sphere-forming cells in the EBV-positive NPC cell line provided us an opportunity to assess the expression of EBV latent and lytic genes in CSC. Notably, elevated expression of EBV latent gene products, EBER1, BARF1 and LMP1 were detected in the CSC subpopulation. We have previously demonstrated that the expression of EBER confers resistance to apoptotic stress [22]. The high EBER expression in NPC CSCs suggests that this cell subpopulation maybe more resistant to apoptosis. On the other hand, LMP1 can induce EMT via Twist or Snail, which coincides with the acquisition of CSC properties [23], [24], [25]. In addition, Kondo *et al*. have also recently shown that LMP1 induces CD44+ CSC in nasopharyngeal epithelial cells [26]. The findings imply that LMP1 expression may play a critical role in CSC maintenance in this EBV-associated malignancy.

In addition to the EBV latent genes, our microarray analysis revealed an overexpression of CCR7 in the NPC-derived spheroids. The enrichment of CCR7+ cell population in the sphere-forming cells was confirmed by flow cytometry. CCR7 is a chemokine receptor that mediates cell migration in response to its ligand CCL21 [27]. It is likely that high CCR7 receptor expression in sphere-forming cells will enhance its sensitivity to the ligands and activate its downstream signaling. Aberrant CCR7 expression in human malignancies has been linked to cell survival and metastatic pathways. The CCL21/CCR7 pathway may play a crucial role in the metastasis of CCR7+ CSCs to lymph nodes [28]. Overexpression of CCR7 in CSCs is likely to contribute to the frequent cervical lymph node metastases in NPC patients [29]. The abolished sphere-forming ability of the NPC cells after CCR7 neutralization suggested CCR7 might also regulate CSC properties in NPC.

Drug resistance is an important characteristic of CSCs, which forms the basis to tumor recurrence after chemotherapeutic treatments [30]. Increased expression of ATP-binding cassette (ABC) transporter family is responsible for the efflux of therapeutic drugs and is a common mechanism for maintaining drug resistance in CSCs [31], [32]. As shown in our microarray study, significant overexpression of ABCC3 and ABCC11 was found in the sphere-forming cells. ABCC3 is a member of the multidrug resistance protein (MRP) subfamily and was shown to be over-expressed in ovarian carcinoma and doxorubicin-resistant lung cancer cell line [33], [34]. ABCC3 overexpression may contribute to the drug resistant properties in the cisplatin-/doxorubicin-resistant C666-1 cell lines (Fig. S5). ABCC11 is another MRP family member up-regulated in NPC-derived spheroids. It is the main transporter for 5-fluorouracil (5-FU) [35] which is one of the main chemotherapeutic drugs for NPC [3]. Over-expression of ABCC11 in sphere-forming cells suggests that the CSC population in NPC is resistant to chemotherapeutic drugs and thus contribute to tumor recurrence.

Hedgehog signaling pathway is an important regulator of CSC maintenance and function. In C666-1 spheroids, the microarray study has also detected the increased expression of GLI1, a major transcription activator of hedgehog signaling pathway. In ovarian carcinoma, GLI1 was reported to regulate the growth of CSCs [36]. To elucidate the role of GLI1 and hedgehog pathway in NPC CSCs, the effects of silencing GLI1 expression on the formation and tumorigenic properties of sphere-forming cells will be examined. The elucidation on signaling mechanisms regulating the proliferation and apoptosis of NPC CSCs will benefit therapies targeted on this subpopulation of drug resistant cells.

In conclusion, we have identified highly tumorigenic CSCs in EBV-positive NPC and this subpopulation could be enriched by cell surface CD44 and identified together with SOX2. Our study has discovered several crucial molecules including CCR7 and ABCC11 involved in the maintenance of NPC CSC functions. The present findings provided a foundation on the development of novel therapies targeting NPC CSC that may potentiate efficacy of current treatments.

## Materials and Methods

### Cell Lines, Xenografts and Primary Tumors

An EBV-positive NPC cell line (C666-1) and 3 xenografts (xeno-666, xeno-2117 and C17) were included in this study [13], [14], [15]. Parental C666-1 was cultured in RPMI-1640 (Sigma-Aldrich) supplemented with 10% fetal bovine serum (FBS) (Invitrogen). For tumor sphere culture, cells were cultured in serum-free DMEM-F12 (Invitrogen) on ultra-low attachment plates. Spheroids were dissociated and passaged every 6–8 days. A total of 1×10^6^ C666-1 cells were seeded per well and the number of spheroids formed was counted.

Five endoscopic tumor biopsies for FACS analysis were obtained from NPC patients with written consents in the Department of Otorhinolaryngology, Prince of Wales Hospital, CUHK. All cases were positive for *EBER*-*in situ* hybridization and histologically diagnosed as undifferentiated or poorly differentiated NPC. We also recruited 49 archival formalin-fixed paraffin-embedded primary tumors from the tissue bank of Department of Anatomical & Cellular Pathology, CUHK for immunohistochemical (IHC) staining. The study protocol was approved by the Clinical Research Ethics Committee of the Chinese University of Hong Kong.

### Quantitative Reverse Transcription and Polymerase Chain Reaction (qRT-PCR) Analysis

Total RNAs were extracted using the Qiagen RNeasy kit (Qiagen) and reverse-transcribed into cDNA using SuperScript cDNA Synthesis Kit (Invitrogen) according to manufacturer protocol. Quantitative RT-PCR were then carried out with specific primers (Invitrogen, primer sequences as listed in Table S1) and Power SYBR® Green PCR mix (Applied Biosystems). β-actin expression was used for data normalization. All qRT-PCRs were performed in triplicates on an ABI 7500 real-time PCR system (Applied Biosystems) as instructed by the manufacturer.

### Fluorescence-activated Cell Sorting (FACS) Analysis

Single cell suspensions obtained from cell lines and tumor tissues were rinsed twice and resuspended in PBS (10^5^ to 10^6^ cells/100 µl). For intracellular staining, cells were fixed in 70% ethanol for 24 hours at −20°C before rehydrating with PBS. Cells were subjected to blocking of non-specific epitopes by 2% human serum and labeled with fluorochrome-conjugated antibodies. Anti-CD44 and anti-CCR7 (BD Pharmingen), and anti-SOX2 (R&D Systems) were used in this study. Respective mouse or rat IgG isotypic controls were included in the experiment. For each sample, at least 10,000 cells were acquired and analyzed with a BD FACSAria flow cytometer (BD Biosciences) and Flowjo software (Treestar).

### Magnetic-activated Cell Sorting (MACS) of CD44+ and CD44− Cells

CD44-positive (CD44+) and CD44-negative (CD44−) C666-1 cells were separated by using anti-CD44 magnetic bead-coupled antibody and the magnetic-activated cell sorting (MACS) system (Miltenyi Biotec). Briefly, cells were incubated with 20 µl of anti-CD44 microbeads (Miltenyi Biotec) per 1×10^7^ cells for 30 minutes at 4°C. Subsequently, cells were washed with PBS supplemented with 0.5% FBS and applied into MACS LS separation columns (Miltenyi Biotec). Purity of CD44+ and CD44− fractions obtained was confirmed by FACS analysis.

### Clone Formation Assay

A total of 1×10^3^ isolated/treated or parental/untreated cells were seeded into 100 mm^2^ plates and cultured for 7–10 days. Cells were then washed with PBS, fixed in methanol for 10 min, and stained with Giemsa stain. Experiments were performed in triplicates and colonies showing size of larger than 50 cells were counted and compared between the two groups.

### Chemoresistant Assays

A total of 5×10^2^ isolated/treated or parental/untreated C666-1 cells were seeded into 96-well plate and cultured for 24 hrs in complete RPMI-1640 medium. The cells were then treated with 0–2.5 µg/mL 5-fluorouracil (5-FU) or non-treated as control as described [37]. The cells were subjected to WST1 cell proliferation assay after 24 hrs of drug treatment. The absorbance measured indicated the proliferation rate of the treated cells and results were compared between CD44+/CD44− or treated/untreated cells.

### In vivo Tumorigenicity Assay in Nude Mice

To evaluate the tumorigenic potential, sphere-forming, monolayer parental C666-1 cells were counted, resuspended, and injected subcutaneously into 4-week-old female nude athymic mice. Mice were inspected daily for tumor formation. After 4 to 12 weeks, mice were sacrificed and the tumors retrieved.

### Microarray Analysis

Total RNA was extracted from sphere-forming and parental C666-1 cells as mentioned above. The Whole Human Genome Oligo Microarray, with over 41000 probes (Agilent Technologies), was used for expression analysis. The expression analysis was carried out according to the manufacturer's protocol. cRNA samples of sphere-forming and parental C666-1 were labeled with 5-CTP (Cy5) and cyanine 3-CTP (Cy-3), respectively. Microarrays were scanned on Agilent’s dual-laser microarray scanner and obtained data were normalized and analyzed using GeneSpring GX version 9.0.5 software (Agilent Technologies). Gene Ontology (GO) analysis of differentially expressed genes was performed by DAVID (The Database for Annotation, Visualization and Integrated Discovery) v6.7 (david.abcc.ncifcrf.gov).

### Immunohistochemistry

For immunohistochemical analysis, anti-CCR7 (Lifespan Biosciences) antibodies were used to determine the CCR7 expression in NPC primary biopsies as previously described [38]. Positive cells were counted and scored according to their prevalence and intensity among tumor cells. The CCR7 expression score was the product of proportion and intensity scores, ranging from 0 to 12. The CCR7 expression was categorized into absence (score 0), low (score 1–3), intermediate (score 4–6), and high (score 7–12). The score was correlated with respective clinical parameters.

### Immunofluorescence Staining

NPC-derived spheroids were transferred onto slide-chambers after 4 weeks of selection and were further cultured for 3 days before fixing with 4% paraformaldehyde for staining. The fixed cells underwent permeabilization with 1% triton-100 in PBS. They were then blocked with 10% rabbit serum in PBS before incubating in anti-human FITC-conjugated CD44 antibody (BD Pharmingen) at 4°C in dark. Excess antibodies were washed away by PBS and then cells were fixed again using 4% paraformaldehyde. Cells were finally mounted in VECTASHIELD® Mounting Medium with DAPI (Vector Laboratories).

### Neutralization of CCR7

To determine the function of CCR7, C666-1 cells were treated with IgG or CCR7 blocking antibody (R&D) and then collected for proliferation and spheroid formation assays.

### Statistical Analysis

All *in vitro* tests were repeated using at least three independent samples for statistical calculations. Unless otherwise stated, unpaired t-test was used for statistical analysis of data. Comparison of IHC scores with clinical data was calculated by Spearman’s correlation. Statistical significance (*P*-value of <0.05) was determined by PRISM5 (Graphpad Software) and presented graphically as mean ± standard error (SE).

## Supporting Information

Figure S1 **CD44 expression in C666-1 spheroids.** CD44 expression was detected on C666-1 spheroids by immunofluorescence staining. CD44 expression was denoted in green by fluorescence FITC while nucleus in blue with DAPI. Negative control was demonstrated by staining with non-specific IgG binding.
(TIF)

Figure S2 **CD133+ cells in NPC.** By flow cytometry, CD133-expressing cells were found to be enriched in sphere-forming C666-1 when compared to NPC cell lines and primary tumors (all *P*<0.001).
(TIF)

Figure S3 **Purity of CD44+ and CD44− cell fractions after MACS separation.** By flow cytometry, CD44 expression was confirmed to be enriched (>70%) in CD44+ cell fractions after MACS separation.
(TIF)

Figure S4 **Correlation of CCR7 and CD44 expression in NPC primary tumors.** A significant linear relationship between CCR7 and CD44 expression was determined in 39 primary cases of NPC (Spearman correlation: r = 0.676, P<0.001)
(TIF)

Figure S5 **Expression of CSC marker CD44 and ABC drug transporters in drug-resistant C666-1.** By qRT-PCR, it was found that CD44+ cells were significantly higher in both cisplatin- and doxorubicin-resistant C666-1 lines than that in parental C666-1. In addition, they were also enriched in ABC transporters ABCC3 and ABCC11. Histograms denoting mean ± SE (n≥3) with statistical significance calculated by t-test (*P<0.05, **P<0.01, ***P<0.001).
(TIF)

Table S1 **Table listing the qPCR primer sequences used in this study.**
(DOCX)

Table S2 **Gene ontology analysis of microarray data.**
(DOCX)

## References

[1] Lo KW, To KF, Huang DP (2004) Focus on nasopharyngeal carcinoma. Cancer Cell 5: 423–428. 15144950 10.1016/s1535-6108(04)00119-9

[2] Pathmanathan R, Prasad U, Chandrika G, Sadler R, Flynn K, et al (1995) Undifferentiated, nonkeratinizing, and squamous cell carcinoma of the nasopharynx. Variants of Epstein-Barr virus-infected neoplasia. Am J Pathol 146: 1355–1367. 7778675 PMC1870892

[3] Chan ATC (2011) Current treatment of nasopharyngeal carcinoma. European Journal of Cancer 47: S302–S303. 21943991 10.1016/S0959-8049(11)70179-4

[4] O'Brien CA, Kreso A, Dick JE (2009) Cancer stem cells in solid tumors: an overview. Semin Radiat Oncol 19: 71–77. 19249644 10.1016/j.semradonc.2008.11.001

[5] Visvader JE (2011) Cells of origin in cancer. Nature 469: 314–322. 21248838 10.1038/nature09781

[6] Curley MD, Therrien VA, Cummings CL, Sergent PA, Koulouris CR, et al (2009) CD133 expression defines a tumor initiating cell population in primary human ovarian cancer. Stem Cells 27: 2875–2883. 19816957 10.1002/stem.236

[7] Joshua B, Kaplan MJ, Doweck I, Pai R, Weissman IL, et al (2012) Frequency of cells expressing CD44, a Head and Neck cancer stem cell marker: Correlation with tumor aggressiveness. Head Neck 34: 42–49. 21322081 10.1002/hed.21699

[8] Liu JC, Deng T, Lehal RS, Kim J, Zacksenhaus E (2007) Identification of tumorsphere- and tumor-initiating cells in HER2/Neu-induced mammary tumors. Cancer Res 67: 8671–8681. 17875707 10.1158/0008-5472.CAN-07-1486

[9] Zhang S, Balch C, Chan MW, Lai HC, Matei D, et al (2008) Identification and characterization of ovarian cancer-initiating cells from primary human tumors. Cancer Res 68: 4311–4320. 18519691 10.1158/0008-5472.CAN-08-0364 PMC2553722

[10] Wang J, Guo LP, Chen LZ, Zeng YX, Lu SH (2007) Identification of cancer stem cell-like side population cells in human nasopharyngeal carcinoma cell line. Cancer Res 67: 3716–3724. 17440084 10.1158/0008-5472.CAN-06-4343

[11] Su J, Xu XH, Huang Q, Lu MQ, Li DJ, et al (2011) Identification of cancer stem-like CD44+ cells in human nasopharyngeal carcinoma cell line. Arch Med Res 42: 15–21. 21376257 10.1016/j.arcmed.2011.01.007

[12] Zhang Y, Peng J, Zhang H, Zhu Y, Wan L, et al (2010) Notch1 signaling is activated in cells expressing embryonic stem cell proteins in human primary nasopharyngeal carcinoma. J Otolaryngol Head Neck Surg 39: 157–166. 20211102 PMC2864547

[13] Cheung ST, Huang DP, Hui AB, Lo KW, Ko CW, et al (1999) Nasopharyngeal carcinoma cell line (C666-1) consistently harbouring Epstein-Barr virus. Int J Cancer 83: 121–126. 10449618 10.1002/(sici)1097-0215(19990924)83:1<121::aid-ijc21>3.0.co;2-f

[14] Busson P, Ganem G, Flores P, Mugneret F, Clausse B, et al (1988) Establishment and characterization of three transplantable EBV-containing nasopharyngeal carcinomas. Int J Cancer 42: 599–606. 2971626 10.1002/ijc.2910420422

[15] Huang DP, Ho JH, Chan WK, Lau WH, Lui M (1989) Cytogenetics of undifferentiated nasopharyngeal carcinoma xenografts from southern Chinese. Int J Cancer 43: 936–939. 2714899 10.1002/ijc.2910430535

[16] Hui AB, Cheung ST, Fong Y, Lo KW, Huang DP (1998) Characterization of a new EBV-associated nasopharyngeal carcinoma cell line. Cancer Genet Cytogenet 101: 83–88. 9494607 10.1016/s0165-4608(97)00231-8

[17] Pastrana E, Silva-Vargas V, Doetsch F (2011) Eyes wide open: a critical review of sphere-formation as an assay for stem cells. Cell Stem Cell 8: 486–498. 21549325 10.1016/j.stem.2011.04.007 PMC3633588

[18] Bussolati B, Bruno S, Grange C, Ferrando U, Camussi G (2008) Identification of a tumor-initiating stem cell population in human renal carcinomas. FASEB J 22: 3696–3705. 18614581 10.1096/fj.08-102590

[19] Leung EL, Fiscus RR, Tung JW, Tin VP, Cheng LC, et al (2010) Non-small cell lung cancer cells expressing CD44 are enriched for stem cell-like properties. PLoS One 5: e14062. 21124918 10.1371/journal.pone.0014062 PMC2988826

[20] Driessens G, Blanpain C (2011) Long live sox2: sox2 lasts a lifetime. Cell Stem Cell 9: 283–284. 21982223 10.1016/j.stem.2011.09.007

[21] Sauvageau M, Sauvageau G (2010) Polycomb group proteins: multi-faceted regulators of somatic stem cells and cancer. Cell Stem Cell 7: 299–313. 20804967 10.1016/j.stem.2010.08.002 PMC4959883

[22] Wong HL, Wang X, Chang RC, Jin DY, Feng H, et al (2005) Stable expression of EBERs in immortalized nasopharyngeal epithelial cells confers resistance to apoptotic stress. Mol Carcinog 44: 92–101. 16086371 10.1002/mc.20133

[23] Horikawa T, Yang J, Kondo S, Yoshizaki T, Joab I, et al (2007) Twist and epithelial-mesenchymal transition are induced by the EBV oncoprotein latent membrane protein 1 and are associated with metastatic nasopharyngeal carcinoma. Cancer Res 67: 1970–1978. 17332324 10.1158/0008-5472.CAN-06-3933

[24] Horikawa T, Yoshizaki T, Kondo S, Furukawa M, Kaizaki Y, et al (2011) Epstein-Barr Virus latent membrane protein 1 induces Snail and epithelial-mesenchymal transition in metastatic nasopharyngeal carcinoma. Br J Cancer 104: 1160–1167. 21386845 10.1038/bjc.2011.38 PMC3068490

[25] Mani SA, Guo W, Liao MJ, Eaton EN, Ayyanan A, et al (2008) The epithelial-mesenchymal transition generates cells with properties of stem cells. Cell 133: 704–715. 18485877 10.1016/j.cell.2008.03.027 PMC2728032

[26] Kondo S, Wakisaka N, Muramatsu M, Zen Y, Endo K, et al (2011) Epstein-barr virus latent membrane protein 1 induces cancer stem/progenitor-like cells in nasopharyngeal epithelial cell lines. J Virol 85: 11255–11264. 21849440 10.1128/JVI.00188-11 PMC3194961

[27] Forster R, Davalos-Misslitz AC, Rot A (2008) CCR7 and its ligands: balancing immunity and tolerance. Nat Rev Immunol 8: 362–371. 18379575 10.1038/nri2297

[28] Li J, Sun R, Tao K, Wang G (2011) The CCL21/CCR7 pathway plays a key role in human colon cancer metastasis through regulation of matrix metalloproteinase-9. Dig Liver Dis 43: 40–47. 20609636 10.1016/j.dld.2010.05.013

[29] Ho FC, Tham IW, Earnest A, Lee KM, Lu JJ (2012) Patterns of regional lymph node metastasis of nasopharyngeal carcinoma: A meta-analysis of clinical evidence. BMC Cancer 12: 98. 22433671 10.1186/1471-2407-12-98 PMC3353248

[30] Garvalov BK, Acker T (2011) Cancer stem cells: a new framework for the design of tumor therapies. J Mol Med (Berl) 89: 95–107. 20890588 10.1007/s00109-010-0685-3

[31] Lou H, Dean M (2007) Targeted therapy for cancer stem cells: the patched pathway and ABC transporters. Oncogene 26: 1357–1360. 17322922 10.1038/sj.onc.1210200

[32] Dean M (2009) ABC transporters, drug resistance, and cancer stem cells. J Mammary Gland Biol Neoplasia 14: 3–9. 19224345 10.1007/s10911-009-9109-9

[33] Auner V, Sehouli J, Oskay-Oezcelik G, Horvat R, Speiser P, et al (2010) ABC transporter gene expression in benign and malignant ovarian tissue. Gynecol Oncol 117: 198–201. 19922990 10.1016/j.ygyno.2009.10.077

[34] Young LC, Campling BG, Cole SP, Deeley RG, Gerlach JH (2001) Multidrug resistance proteins MRP3, MRP1, and MRP2 in lung cancer: correlation of protein levels with drug response and messenger RNA levels. Clin Cancer Res 7: 1798–1804. 11410522

[35] Oguri T, Bessho Y, Achiwa H, Ozasa H, Maeno K, et al (2007) MRP8/ABCC11 directly confers resistance to 5-fluorouracil. Mol Cancer Ther 6: 122–127. 17237272 10.1158/1535-7163.MCT-06-0529

[36] Mine T, Matsueda S, Gao H, Li Y, Wong KK, et al (2010) Created Gli-1 duplex short-RNA (i-Gli-RNA) eliminates CD44Hi progenitors of taxol-resistant ovarian cancer cells. Oncol Rep 23: 1537–1543. 20428807 10.3892/or_00000793 PMC3426036

[37] Chia MC, Leung A, Krushel T, Alajez NM, Lo KW, et al (2008) Nuclear factor-Y and Epstein Barr virus in nasopharyngeal cancer. Clin Cancer Res 14: 984–994. 18281530 10.1158/1078-0432.CCR-07-0828

[38] Kang W, Tong JH, Chan AW, Lung RW, Chau SL, et al (2012) Stathmin1 plays oncogenic role and is a target of microRNA-223 in gastric cancer. PLoS One 7: e33919. 22470493 10.1371/journal.pone.0033919 PMC3314670

