# Supplementary figures and images for: CD44+ Cancer Stem-Like Cells in EBV-Associated Nasopharyngeal Carcinoma
Source: PLoS One. 2012 Dec 21;7(12):e52426. doi: 10.1371/journal.pone.0052426 (PMC3528656; doi:10.1371/journal.pone.0052426)

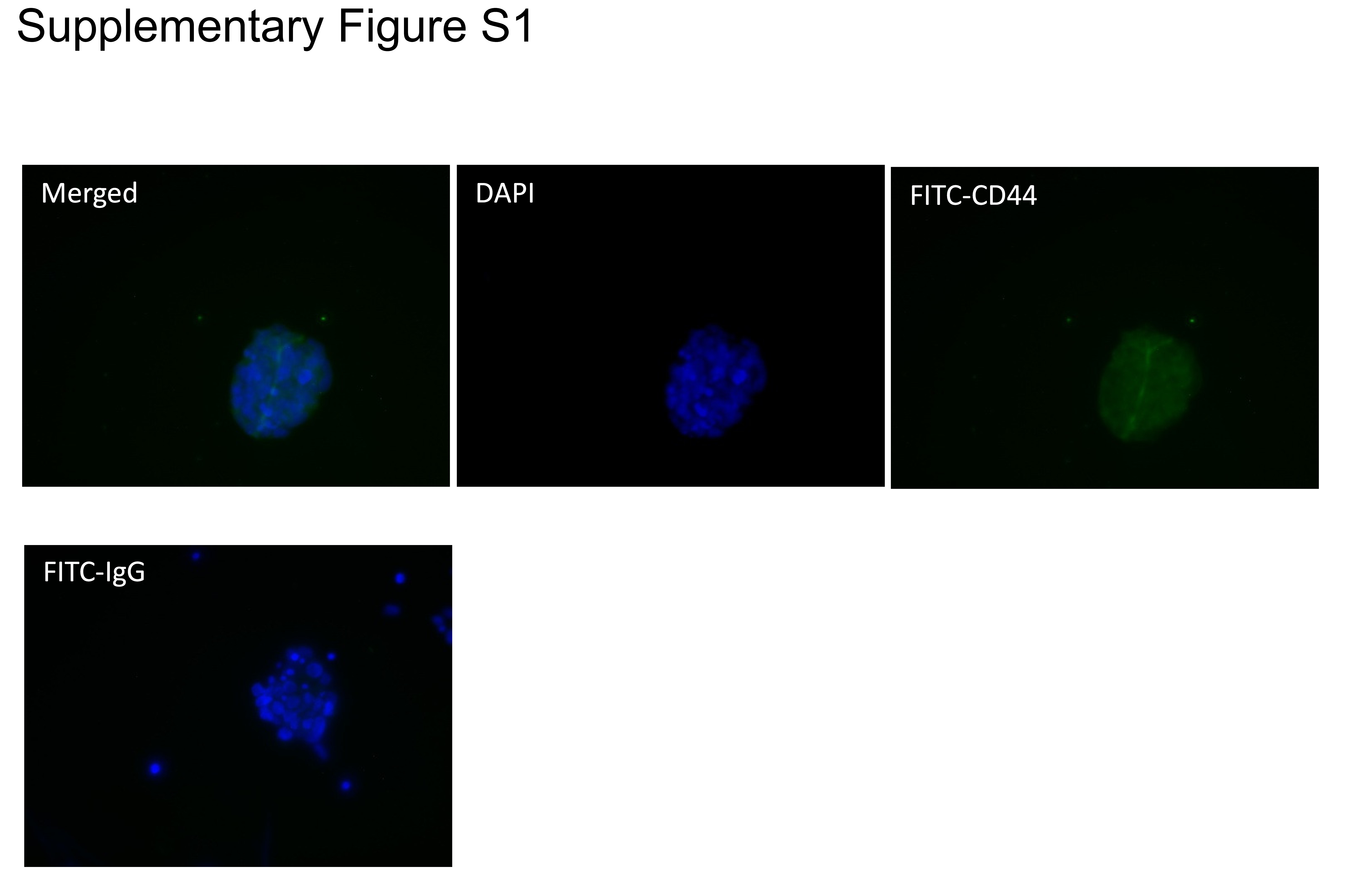

Supplement: Figure S1 — CD44 expression in C666-1 spheroids. CD44 expression was detected on C666-1 spheroids by immunofluorescence staining. CD44 expression was denoted in green by fluorescence FITC while nucleus in blue with DAPI. Negative control was demonstrated by staining with non-specific IgG binding. (TIF) [file pone.0052426.s001.tif]

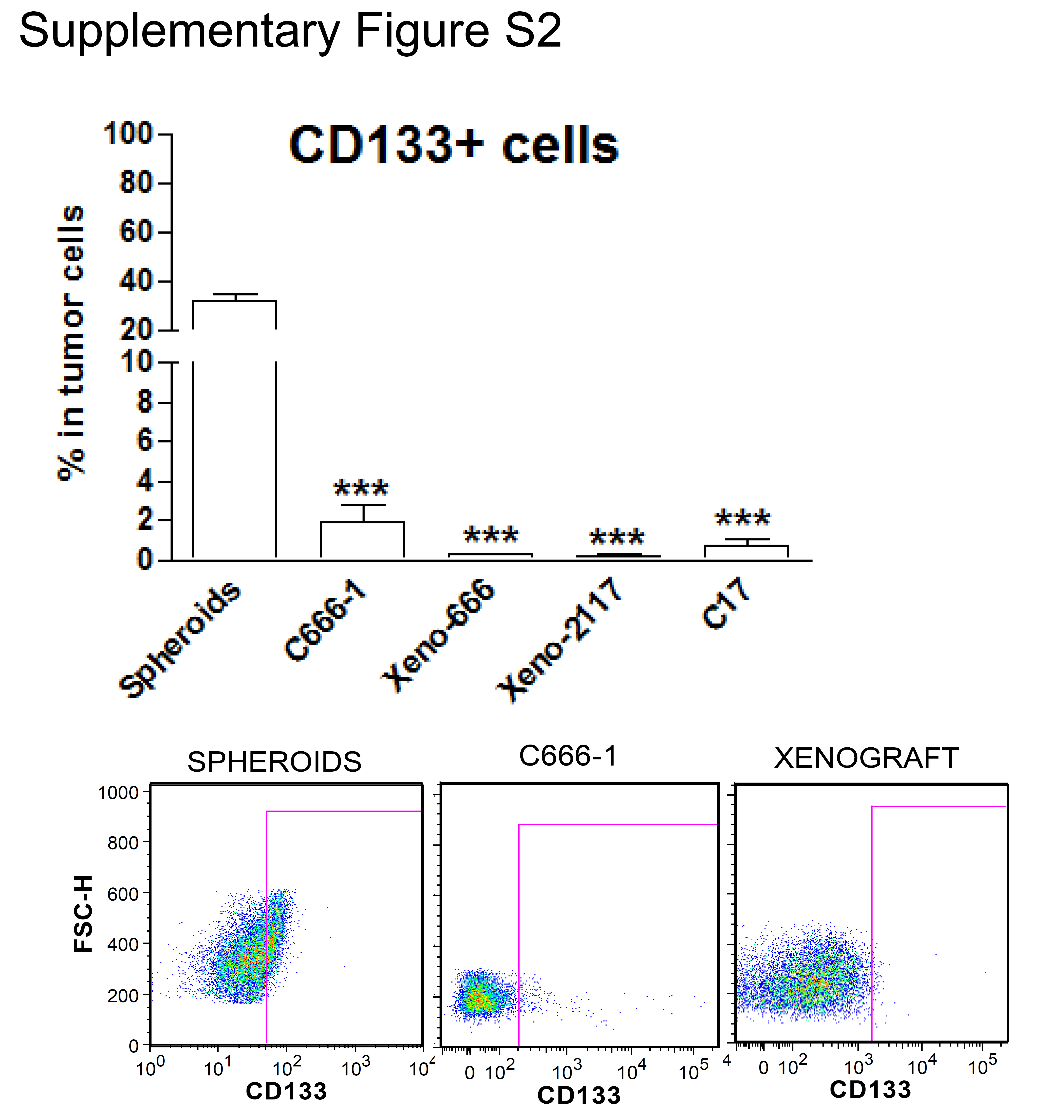

Supplement: Figure S2 — CD133+ cells in NPC. By flow cytometry, CD133-expressing cells were found to be enriched in sphere-forming C666-1 when compared to NPC cell lines and primary tumors (all P<0.001). (TIF) [file pone.0052426.s002.tif]

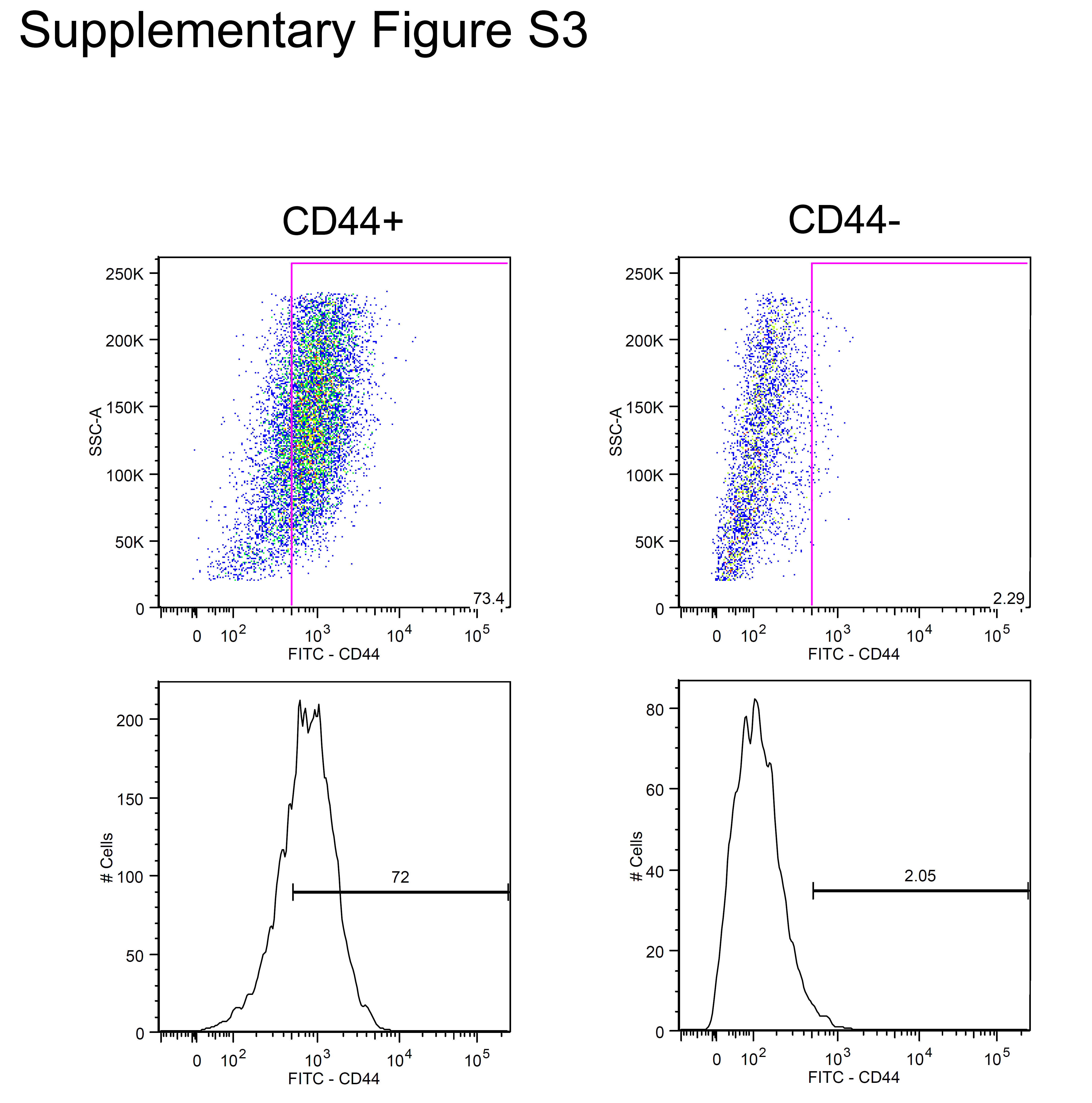

Supplement: Figure S3 — Purity of CD44+ and CD44− cell fractions after MACS separation. By flow cytometry, CD44 expression was confirmed to be enriched (>70%) in CD44+ cell fractions after MACS separation. (TIF) [file pone.0052426.s003.tif]

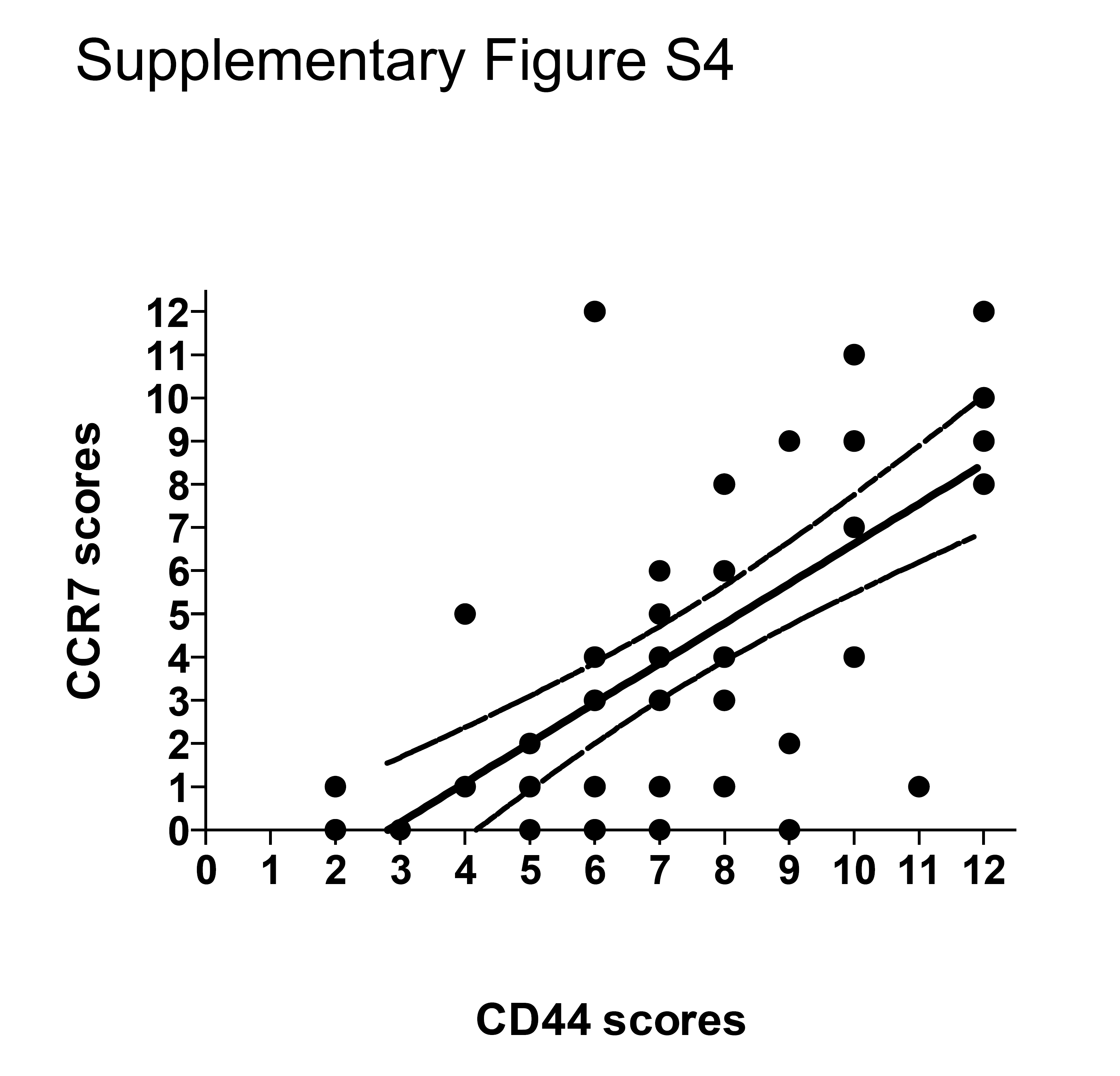

Supplement: Figure S4 — Correlation of CCR7 and CD44 expression in NPC primary tumors. A significant linear relationship between CCR7 and CD44 expression was determined in 39 primary cases of NPC (Spearman correlation: r = 0.676, P<0.001) (TIF) [file pone.0052426.s004.tif]

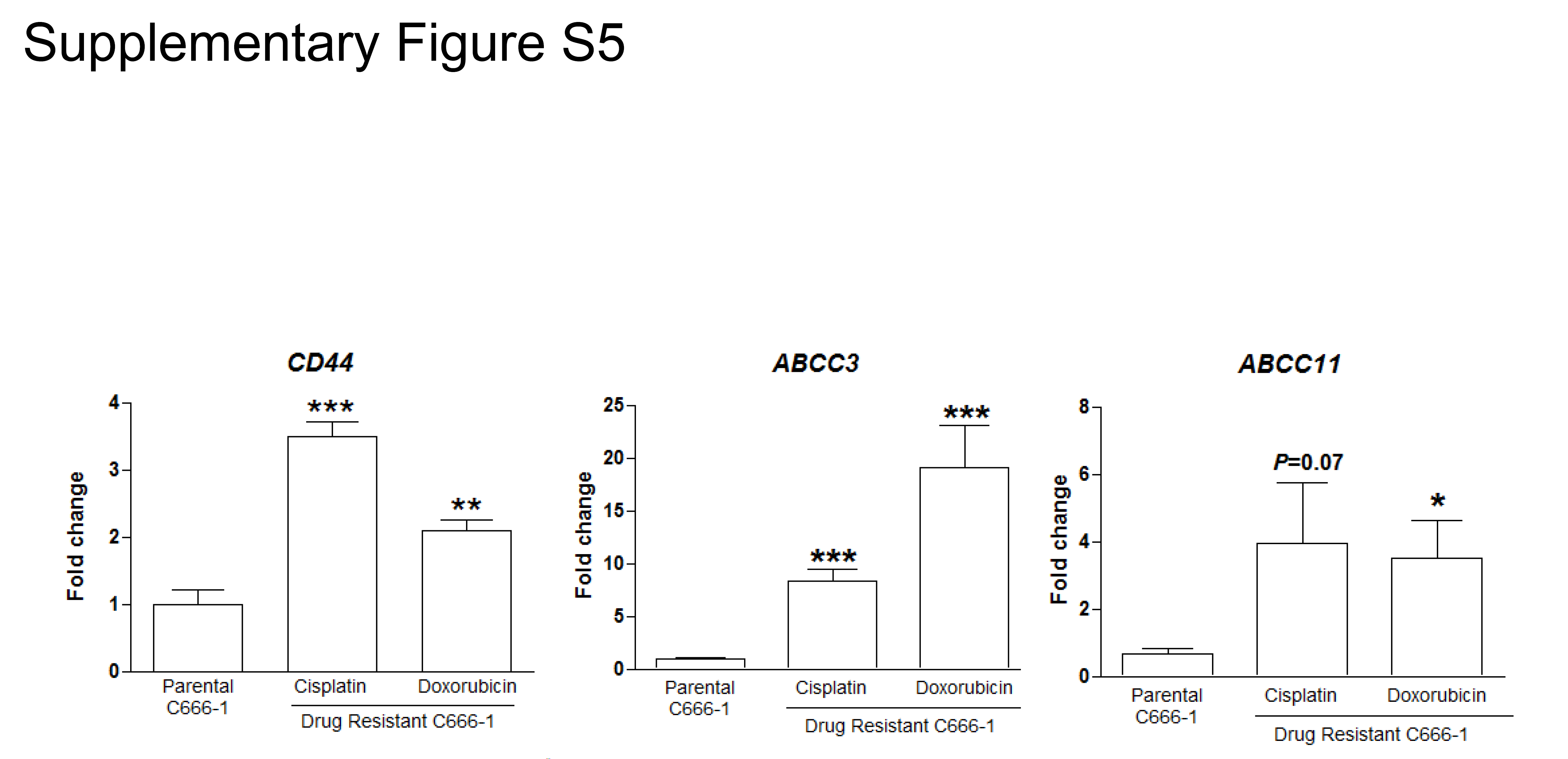

Supplement: Figure S5 — Expression of CSC marker CD44 and ABC drug transporters in drug-resistant C666-1. By qRT-PCR, it was found that CD44+ cells were significantly higher in both cisplatin- and doxorubicin-resistant C666-1 lines than that in parental C666-1. In addition, they were also enriched in ABC transporters ABCC3 and ABCC11. Histograms denoting mean ± SE (n≥3) with statistical significance calculated by t-test (*P<0.05, **P<0.01, ***P<0.001). (TIF) [file pone.0052426.s005.tif]
