# Supplementary material for: CD44+ Cancer Stem-Like Cells in EBV-Associated Nasopharyngeal Carcinoma
Source: PLoS One. 2012 Dec 21;7(12):e52426. doi: 10.1371/journal.pone.0052426 (PMC3528656; doi:10.1371/journal.pone.0052426)
Supplement: Table S1 — Table listing the qPCR primer sequences used in this study. (DOCX) [file pone.0052426.s006.docx]

### Supplementary Table S1. Table listing the qPCR primer sequences used in this study.

| β-ACTIN | 5’ - GTCTTCCCCTCCATCGTG - 3’ |
| --- | --- |
|  | 5’ - AGGGTGAGGATGCCTCTCTT - 3’ |
| OCT-4 | 5’ - TGGAGAAGGAGAAGCTGGAGCAAAA - 3’ |
|  | 5’ - GGCAGATGGTCGTTTGGCTGAATA - 3’ |
| NANOG | 5’ - GATTTGTGGGCCTGAAGAAA - 3’ |
|  | 5’ - AAGTGGGTTGTTTGCCTTTG - 3’ |
| ALDH1 | 5’ - TCCTGGTTATGGGCCTACAG - 3’ |
|  | 5’ - CTGGCCCTGGTGGTAGAATA - 3’ |
| CKIT | 5’ - ACCATTCTGTGCGGATCAAT - 3’ |
|  | 5’ - GACAAAAATCATCGGCCACT - 3’ |
| SOX2 | 5’ - TACAGCATGTCCTACTCGCAG - 3’ |
|  | 5’ - GAGGAAGAGGTAACCACAGGG - 3’ |
| BMI1 | 5’ - CCAGGGCTTTTCAAAAATGA - 3’ |
|  | 5’ - CCGATCCAATCTGTTCTGGT - 3’ |
| KLF4 | 5’ - GGACATCAACGACGTGAGC - 3’ |
|  | 5’ - GACGCCTTCAGCACGAAC - 3’ |
| KLF5 | 5’ - GGTTGCACAAAAGTTTATAC - 3’ |
|  | 5’ - GGCTTGGCGCCTGTGTGCTTCC - 3’ |
| CD44 | 5’ - TCAGAGGAGTAGGAGAGAGGAAAC - 3’ |
|  | 5’ - GAAAAGTCAAAGTAACAATAACAGTGG - 3’ |
| CD133 | 5’ - GCATTGGCATCTTCTATGGTT - 3’ |
|  | 5’ - CGCCTTGTCCTTGGTAGTGT - 3’ |
| NESTIN | 5’ - AACAGCGACGGAGGTCTCTA - 3’ |
|  | 5’ - TTCTCTTGTCCCGCAGACTT - 3’ |
| ABCB1 | 5’ - CTCATGATGCTGGTGTTTGG - 3’ |
|  | 5’ - TGGTCATGTCTTCCTCCAGA - 3’ |
| ABCC1 | 5’ - CGGATGTCATCTGAAATGGGA - 3’ |
|  | 5’ - GAGCTGTCTCCTGGATTTGC - 3’ |
| CYP2C8 | 5’ - TGTGGTCCTGGTGCTGTG - 3’ |
|  | 5’ - ATATTGGGGAATTGCCTCTT - 3’ |
| BZLF1 | 5’ – AACTCCATGTCCTTCCAACG – 3’ |
|  | 5’ – GTGGGGGAATATGGGTCTCT – 3’ |
| BARF1 | 5’ – AGGTCACCAAGCAGGAACAC – 3’ |
|  | 5’ – CGGTGCATGTCACAGTAAGG – 3’ |
| EBERs | 5’ - AGGACCTACGCTGCCCTAGA - 3’ |
|  | 5’ - AAAACATGCGGACCACCAGC - 3’ |
| EBNA1 | 5’ - CCTACAGGGTGGAAAAATGGC - 3’ |
|  | 5’ - TCATCATCATCCGGGTCTCC - 3’ |
| LMP1 | 5’ - CAGTCAGGCAAGCCTATGA - 3’ |
|  | 5’ - CTGGTTCCGGTGGAGATGA - 3’ |
| LMP2A | 5’ - AGCTGTAACTGTGGTTTCCATGAC - 3’ |
|  | 5’ - GCCCCCTGGCGAAGAG - 3’ |
| IL-8 | 5’ - TAGCAAAATTGAGGCCAAGG - 3’ |
|  | 5’ - AAACCAAGGCACAGTGGAAC - 3’ |
| SELE | 5’ - AGCCCAGAGCCTTCAGTGTA - 3’ |
|  | 5’ - AACTGGGATTTGCTGTGTCC - 3’ |
| GLI1 | 5’ - GAACCTTCCTACCAGAGTCC - 3’ |
|  | 5’ - GTGCTGCTGCCCTATGTG - 3’ |
| FOXN4 | 5’ - AAGAACTGCACCCCAAACAC - 3’ |
|  | 5’ - GAGCCGCTCATCTTGTTCTC - 3’ |
| ABCC3 | 5’ - TTTTCTTTGTCACCCCCTTG - 3’ |
|  | 5’ - AAAGGATCTTGGAGCGGAAT - 3’ |
| ABCC11 | 5’ - CATCAGCTCACACAGGAGGA - 3’ |
|  | 5’ - CCTGCTCCAACCAGTAGCTC - 3’ |
