# Supplementary material for: CD44+ Cancer Stem-Like Cells in EBV-Associated Nasopharyngeal Carcinoma
Source: PLoS One. 2012 Dec 21;7(12):e52426. doi: 10.1371/journal.pone.0052426 (PMC3528656; doi:10.1371/journal.pone.0052426)
Supplement: Table S2 — Gene ontology analysis of microarray data. (DOCX) [file pone.0052426.s007.docx]

**Supplementary Table S2. Gene ontology analysis of microarray data**

| **Gene Symbol** | **Gene Description (all genes of Homo sapiens)** | **Genbank Accession no.** | **Fold change** |
| --- | --- | --- | --- |
|  | | | |
| **Regulation Transcription** | | | |
| FOXN4 | Forkhead box N4 | NM_213596 | 43.28 |
| IL6 | Interleukin 6 (interferon, beta 2) | NM_000600 | 43.24 |
| EGR1 | Early growth response 1 | NM_001964 | 33.48 |
| ZNF452 | Zinc finger protein 452 | NM_052923 | 28.68 |
| ZNF514 | Zinc finger protein 514 | NM_032788 | 22.04 |
| DLX1 | Distal-less homeobox 1, transcript variant 1 | NM_178120 | 20.71 |
| HOXA7 | Homeobox A7 | NM_006896 | 20.43 |
| KCNIP3 | Kv channel interacting protein 3, calsenilin, transcript variant 1 | NM_013434 | 20.11 |
| SP100 | Nuclear autoantigen | L79989 | 19.64 |
| ZNF563 | Zinc finger protein 563 | NM_145276 | 16.96 |
| HOMEZ | Homeobox and leucine zipper encoding | NM_020834 | 16.71 |
| ZNF70 | Zinc finger protein 70 | NM_021916 | 16.51 |
| FHL2 | Four and a half LIM domains 2, transcript variant 2 | NM_201555 | 16.45 |
| ZNF229 | Zinc finger protein 229 | NM_014518 | 16.06 |
| ZNF75 | Zinc finger protein 75 (D8C6) | NM_007131 | 15.53 |
| TBX4 | T-box 4 | NM_018488 | 15.50 |
| ZNF311 | Zinc finger protein 311 | NM_001010877 | 15.09 |
| ZNF181 | Zinc finger protein 181 | NM_001029997 | 14.78 |
| ZSCAN4 | Zinc finger and SCAN domain containing 4 | NM_152677 | 14.64 |
| TSHZ2 | Teashirt family zinc finger 2 | NM_173485 | 13.10 |
| ZNF302 | Zinc finger protein 302, transcript variant 1 | NM_018443 | 12.97 |
| ZNF658 | Zinc finger protein 658 | NM_033160 | 12.47 |
| EGR2 | Early growth response 2 | NM_000399 | 12.17 |
| GLIS3 | GLIS family zinc finger 3, transcript variant 1 | NM_001042413 | 12.00 |
| STAT4 | Signal transducer and activator of transcription 4 | NM_003151 | 11.99 |
| AK001503 | cDNA FLJ10641 fis, clone NT2RP2005748 | AK001503 | 11.58 |
| ZNF429 | Zinc finger protein 429 | NM_001001415 | 11.31 |
| ZNF75A | Zinc finger protein 75a | NM_153028 | 10.72 |
| TRAK2 | Trafficking protein, kinesin binding 2 | NM_015049 | 10.52 |
| BCL11B | B-cell CLL/lymphoma 11B (zinc finger protein), transcript variant 1 | NM_138576 | 10.52 |
| ZNF347 | Zinc finger protein 347 | NM_032584 | 10.36 |
| ZNF41 | Zinc finger protein 41, transcript variant 2 | NM_153380 | 9.83 |
| ZNF425 | Zinc finger protein 425 | NM_001001661 | 9.44 |
| KLF15 | Kruppel-like factor 15 | NM_014079 | 9.40 |
| ZNF490 | Zinc finger protein 490 | NM_020714 | 9.36 |
| ZNF547 | Zinc finger protein 547 | NM_173631 | 9.20 |
| NFATC1 | Nuclear factor of activated T-cells, cytoplasmic, calcineurin-dependent 1, transcript variant 3 | NM_172387 | 8.97 |
| AHRR | Aryl-hydrocarbon receptor repressor | NM_020731 | 8.88 |
| GLI1 | Glioma-associated oncogene homolog 1 (zinc finger protein) | NM_005269 | 8.84 |
| ZNF616 | cDNA: FLJ21781 fis, clone HEP00223 | AK025434 | 8.82 |
| ZNF500 | mRNA for KIAA0557 protein, partial cds | AB011129 | 8.81 |
| BATF2 | Basic leucine zipper transcription factor, ATF-like 2 | NM_138456 | 8.68 |
| ZNF37A | Zinc finger protein 37A, transcript variant 1 | NM_001007094 | 8.40 |
| FOXD4 | Forkhead box D4 | NM_207305 | 8.35 |
| HCLS1 | Hematopoietic cell-specific Lyn substrate 1 | NM_005335 | 8.32 |
| ZNF189 | Zinc finger protein 189, transcript variant 2 | NM_197977 | 8.19 |
| TFAP2B | Transcription factor AP-2 beta (activating enhancer binding protein 2 beta) | NM_003221 | 8.02 |
| DENND4A | mRNA; cDNA DKFZp313L197 (from clone DKFZp313L197) | AL833317 | 7.92 |
| ZNF556 | Zinc finger protein 556 | NM_024967 | 7.84 |
| ARID5B | AT rich interactive domain 5B (MRF1-like) | NM_032199 | 7.72 |
| GATAD2A | GATA zinc finger domain containing 2A | AK024670 | 7.57 |
| ZNF596 | Zinc finger protein 596, transcript variant 3 | NM_173539 | 7.51 |
| AFAP1L2 | Actin filament associated protein 1-like 2, transcript variant 1 | NM_001001936 | 7.49 |
| ZBTB32 | Zinc finger and BTB domain containing 32 | NM_014383 | 7.44 |
| ZNF780A | cDNA FLJ33955 fis, clone CTONG2018652, moderately similar to ZINC FINGER PROTEIN MFG-3 | AK091274 | 7.40 |
| ZNF23 | Clone L3-15 zinc finger protein mRNA, partial cds | AF024709 | 7.27 |
| HDAC4 | Histone deacetylase 4 | NM_006037 | 7.13 |
| CXorf43 | Chromosome X open reading frame 43 | NM_144657 | 7.08 |
| DNAJB6 | DnaJ (Hsp40) homolog, subfamily B, member 6, transcript variant 1 | NM_058246 | 7.07 |
| BATF | Basic leucine zipper transcription factor, ATF-like | NM_006399 | 6.97 |
| ZNF382 | Zinc finger protein 382 | NM_032825 | 6.94 |
| PRDM7 | PR domain containing 7 | NM_052996 | 6.59 |
| ZNF19 | Zinc finger protein 19 | NM_006961 | 6.55 |
| ZFHX2 | mRNA for KIAA1762 protein, partial cds | AB051549 | 6.54 |
| ZSCAN5 | Zinc finger and SCAN domain containing 5 | NM_024303 | 6.47 |
| ALS2CR8 | Amyotrophic lateral sclerosis 2 (juvenile) chromosome region, candidate 8 | NM_024744 | 6.41 |
| ZNF160 | Zinc finger protein 160, transcript variant 2 | NM_198893 | 6.39 |
| ZNF549 | Zinc finger protein 549 | NM_153263 | 6.27 |
| ZNF268 | HZF3 mRNA for zinc finger protein | X78926 | 6.16 |
| ZNF396 | Zinc finger protein | AF533251 | 6.13 |
| ZNF251 | Zinc finger protein 251, cDNA clone IMAGE:3948563, partial cds | BC006258 | 6.08 |
| ZNF461 | Gonadotropin inducible transcription repressor 1 | NM_153257 | 6.05 |
| ZNF180 | Zinc finger protein 180 | NM_013256 | 6.04 |
| INHBA | Inhibin, beta A (activin A, activin AB alpha polypeptide) | NM_002192 | 6.02 |
| TRIM22 | Tripartite motif-containing 22 | NM_006074 | 5.97 |
| ZNF761 | Zinc finger protein 761 | NM_001008401 | 5.77 |
| ZNF510 | Zinc finger protein 510 | NM_014930 | 5.59 |
| ZFP90 | Zinc finger protein 90 homolog (mouse) | NM_133458 | 5.47 |
| ZNF718 | Zinc finger protein 718 | NM_001039127 | 5.42 |
| HEYL | Hairy/enhancer-of-split related with YRPW motif-like | NM_014571 | 5.38 |
| ZNF485 | Zinc finger protein 485 | NM_145312 | 5.33 |
| HOXB8 | Homeobox B8 | NM_024016 | 5.30 |
| ZNF440 | cDNA FLJ35240 fis, clone PROST2002425, moderately similar to ZINC FINGER PROTEIN 136 | AK092559 | 5.29 |
| ADRB2 | Adrenergic, beta-2-, receptor, surface | NM_000024 | 5.26 |
| RUNX1 | Runt-related transcription factor 1 (acute myeloid leukemia 1; aml1 oncogene), transcript variant 2 | NM_001001890 | 5.25 |
| PCBD2 | Pterin-4 alpha-carbinolamine dehydratase/dimerization cofactor of hepatocyte nuclear factor 1 alpha (TCF1) 2 | NM_032151 | 5.22 |
| ZNF232 | Zinc finger protein 232 | NM_014519 | 5.20 |
| ZNF416 | Zinc finger protein 416 | NM_017879 | 5.20 |
| MAF | v-maf musculoaponeurotic fibrosarcoma oncogene homolog (avian), transcript variant 1 | NM_005360 | 5.11 |
| KCNH4 | Potassium voltage-gated channel, subfamily H (eag-related), member 4 | NM_012285 | 5.10 |
| TCF1 | Transcription factor 1, hepatic; LF-B1, hepatic nuclear factor (HNF1), albumin proximal factor | NM_000545 | 5.07 |
| ZNF214 | Zinc finger protein 214 | NM_013249 | 5.00 |
| ZNF45 | Zinc finger protein 45 | NM_003425 | 5.00 |
| MBD2 | Methyl-CpG binding domain protein 2, transcript variant testis-specific | NM_015832 | -22.31 |
| STAT3 | Signal transducer and activator of transcription 3 (acute-phase response factor), mRNA (cDNA clone IMAGE:5193339), partial cds | BC029783 | -16.81 |
| CREB3L2 | cAMP responsive element binding protein 3-like 2, mRNA (cDNA clone IMAGE:4185677), complete cds | BC063666 | -13.42 |
| KLF11 | Kruppel-like factor 11 | NM_003597 | -11.23 |
| MYCL1 | v-myc myelocytomatosis viral oncogene homolog 1, lung carcinoma derived (avian), transcript variant 3 | NM_005376 | -10.80 |
| JMY | Junction-mediating and regulatory protein | NM_152405 | -10.77 |
| NR1D1 | Nuclear receptor subfamily 1, group D, member 1 | NM_021724 | -10.72 |
| CNOT2 | mRNA; cDNA DKFZp434M0572 (from clone DKFZp434M0572); partial cds | AL137674 | -10.63 |
| ZNF367 | Zinc finger protein 367 | NM_153695 | -9.03 |
| ABL1 | v-abl Abelson murine leukemia viral oncogene homolog 1, transcript variant b | NM_007313 | -9.03 |
| SERTAD2 | SERTA domain containing 2 | NM_014755 | -8.60 |
| SOD2 | Superoxide dismutase 2, mitochondrial, mRNA (cDNA clone MGC:21350 IMAGE:4184203), complete cds | BC016934 | -8.39 |
| PAX6 | Paired box gene 6 (aniridia, keratitis), transcript variant 2 | NM_001604 | -7.70 |
| ZNF174 | Truncated zinc finger protein isoform | AF542096 | -7.62 |
| NCOR1 | Nuclear receptor co-repressor 1 | NM_006311 | -7.56 |
| PER1 | Period homolog 1 (Drosophila) | NM_002616 | -7.25 |
| PER3 | Period homolog 3 (Drosophila) | NM_016831 | -7.14 |
| TCF25 | Transcription factor 25 (basic helix-loop-helix) | NM_014972 | -7.08 |
| BRF1 | BRF1 homolog, subunit of RNA polymerase III transcription initiation factor IIIB (S. cerevisiae), mRNA (cDNA clone IMAGE:4830425), complete cds | BC016743 | -7.07 |
| RASD1 | RAS, dexamethasone-induced 1 | NM_016084 | -7.05 |
| CREBBP | CREB binding protein (Rubinstein-Taybi syndrome), transcript variant 1 | NM_004380 | -6.75 |
| HSF2 | Heat shock transcription factor 2 | NM_004506 | -6.56 |
| ZNF175 | Zinc finger protein 175, mRNA (cDNA clone IMAGE:4301632), partial cds | BC007778 | -6.53 |
| DBP | D site of albumin promoter (albumin D-box) binding protein | NM_001352 | -6.21 |
| CHD2 | Chromodomain helicase DNA binding protein 2, transcript variant 1 | NM_001271 | -6.19 |
| MED9 | Mediator of RNA polymerase II transcription, subunit 9 homolog (S. cerevisiae), mRNA (cDNA clone IMAGE:4095249) | BC010906 | -6.15 |
| HHEX | Hematopoietically expressed homeobox | NM_002729 | -5.93 |
| SIX4 | Sine oculis homeobox homolog 4 (Drosophila) | NM_017420 | -5.87 |
| ZNF224 | Zinc finger protein 224, mRNA (cDNA clone IMAGE:3941350), partial cds | BC002889 | -5.78 |
| IRX4 | Iroquois homeobox protein 4 | NM_016358 | -5.74 |
| HDAC6 | Histone deacetylase 6, mRNA (cDNA clone IMAGE:4179066), complete cds | BC011498 | -5.71 |
| ZNF286A | Full length insert cDNA clone ZD50H12 | AF086305 | -5.52 |
| MYBL1 | v-myb myeloblastosis viral oncogene homolog (avian)-like 1 | NM_001080416 | -5.34 |
| TLE3 | Transducin-like enhancer of split 3 (E(sp1) homolog, Drosophila) | NM_005078 | -5.24 |
| RXRA | Retinoid X receptor, alpha | NM_002957 | -5.24 |
| PSRC1 | Proline/serine-rich coiled-coil 1, transcript variant 1 | NM_032636 | -5.17 |
| KLF7 | Kruppel-like factor 7 (ubiquitous) | NM_003709 | -5.15 |
| MDM4 | Mdm4, transformed 3T3 cell double minute 4, p53 binding protein (mouse) | NM_002393 | -5.05 |
|  | | | |
| **Immune response** | | | |
| CCR7 | Chemokine (C-C motif) receptor 7 | NM_001838 | 169.42 |
| IL6 | Interleukin 6 (interferon, beta 2) | NM_000600 | 43.24 |
| AQP9 | Aquaporin 9 | NM_020980 | 23.50 |
| IL1B | Interleukin 1, beta | NM_000576 | 22.54 |
| CCL4 | Chemokine (C-C motif) ligand 4, transcript variant 1 | NM_002984 | 20.85 |
| SP100 | Nuclear autoantigen mRNA, partial cds; alternatively spliced | L79989 | 19.64 |
| RAG1 | Recombination activating gene 1 | NM_000448 | 16.95 |
| CCL2 | Chemokine (C-C motif) ligand 2 | NM_002982 | 14.40 |
| IL7R | Interleukin 7 receptor | NM_002185 | 13.75 |
| KRT1 | Keratin 1 (epidermolytic hyperkeratosis) | NM_006121 | 10.49 |
| TNFSF13B | Tumor necrosis factor (ligand) superfamily, member 13b | NM_006573 | 10.10 |
| FYB | FYN binding protein (FYB-120/130), transcript variant 1 | NM_001465 | 9.91 |
| CX3CL1 | Chemokine (C-X3-C motif) ligand 1 | NM_002996 | 9.49 |
| NOD2 | Nucleotide-binding oligomerization domain containing 2 | NM_022162 | 8.70 |
| IGKV1-5 | Immunoglobulin kappa variable 1-5, mRNA (cDNA clone MGC:32715 IMAGE:4694346), complete cds | BC034142 | 8.28 |
| CCL3 | mRNA for pLD78 peptide, complete cds | D00044 | 8.22 |
| LCP2 | Lymphocyte cytosolic protein 2 (SH2 domain containing leukocyte protein of 76kDa) | NM_005565 | 8.11 |
| IL8 | Interleukin 8 | NM_000584 | 8.00 |
| SARM1 | Sterile alpha and TIR motif containing 1 | NM_015077 | 7.95 |
| CCL3L3 | Chemokine (C-C motif) ligand 3-like 3 | NM_001001437 | 7.84 |
| CD86 | CD86 molecule, transcript variant 2 | NM_006889 | 7.78 |
| CNGA1 | Cyclic nucleotide gated channel alpha 1 | NM_000087 | 7.35 |
| CCL25 | Chemokine (C-C motif) ligand 25 | NM_005624 | 7.27 |
| KYNU | Kynureninase (L-kynurenine hydrolase), transcript variant 1 | NM_003937 | 7.06 |
| GBP7 | Guanylate binding protein 7 | NM_207398 | 7.04 |
| MR1 | Major histocompatibility complex, class I-related | NM_001531 | 6.74 |
| TREM2 | Triggering receptor expressed on myeloid cells 2 | NM_018965 | 6.64 |
| SLAMF7 | SLAM family member 7 | NM_021181 | 6.47 |
| CCL17 | Chemokine (C-C motif) ligand 17 | NM_002987 | 6.44 |
| TRIM22 | Tripartite motif-containing 22 | NM_006074 | 5.97 |
| ENPP3 | Ectonucleotide pyrophosphatase/phosphodiesterase 3 | NM_005021 | 5.94 |
| CARD9 | Caspase recruitment domain family, member 9 | NM_052813 | 5.88 |
| NOS2A | Nitric oxide synthase 2A (inducible, hepatocytes), transcript variant 1 | NM_000625 | 5.87 |
| IL20RB | Interleukin 20 receptor beta | NM_144717 | 5.83 |
| POU2AF1 | POU domain, class 2, associating factor 1 | NM_006235 | 5.76 |
| ST6GAL1 | ST6 beta-galactosamide alpha-2,6-sialyltranferase 1, transcript variant 1 | NM_173216 | 5.68 |
| TREML1 | Triggering receptor expressed on myeloid cells-like 1 | NM_178174 | 5.66 |
| CD8A | CD8a molecule, transcript variant 1 | NM_001768 | 5.48 |
| TNFRSF17 | Tumor necrosis factor receptor superfamily, member 17 | NM_001192 | 5.47 |
| CCL13 | Chemokine (C-C motif) ligand 13 | NM_005408 | 5.32 |
| TLR7 | Toll-like receptor 7 | NM_016562 | 5.28 |
| C8G | Complement component 8, gamma polypeptide | NM_000606 | 5.23 |
| VAV1 | Vav 1 oncogene | NM_005428 | 5.18 |
| DMBT1 | Deleted in malignant brain tumors 1, transcript variant 2 | NM_007329 | 5.05 |
| PVRL1 | Poliovirus receptor-related 1 (herpesvirus entry mediator C; nectin), transcript variant 3 | NM_203286 | -10.73 |
| CD55 | Decay-accelerating factor 4ab mRNA, partial cds, alternatively spliced | AY055760 | -9.44 |
| PRG3 | Proteoglycan 3 | NM_006093 | -8.50 |
| LILRB5 | Leukocyte immunoglobulin-like receptor, subfamily B (with TM and ITIM domains), member 5, transcript variant 2 | NM_006840 | -8.14 |
| HLA-G | HLA-G histocompatibility antigen, class I, G | NM_002127 | -6.94 |
| APOA4 | Apolipoprotein A-IV | NM_000482 | -6.91 |
| SWAP70 | SWAP-70 protein, mRNA (cDNA clone IMAGE:2900736), containing frame-shift errors | BC000134 | -6.25 |
| MICA | MHC class I polypeptide-related sequence A | NM_000247 | -5.73 |
| GTPBP1 | GTP binding protein 1 | NM_004286 | -5.55 |
| IGF1R | Clone 1900 unknown protein mRNA, complete cds | AF020763 | -5.47 |
|  | | | |
| **Cell adhesion** | | | |
| SELE | Selectin E (endothelial adhesion molecule 1) | NM_000450 | 149.51 |
| CCL4 | Chemokine (C-C motif) ligand 4, transcript variant 1 | NM_002984 | 20.85 |
| AMBP | Alpha-1-microglobulin/bikunin precursor | NM_001633 | 16.44 |
| CDH26 | Cadherin-like 26 (CDH26), transcript variant b | NM_021810 | 15.50 |
| FCGBP | Fc fragment of IgG binding protein | NM_003890 | 14.90 |
| TNC | Tenascin C (hexabrachion) | NM_002160 | 14.62 |
| CCL2 | Chemokine (C-C motif) ligand 2 | NM_002982 | 14.40 |
| BCAN | Brevican, mRNA (cDNA clone IMAGE:3618761), partial cds | BC005081 | 14.00 |
| CDH11 | Cadherin 11, type 2, OB-cadherin (osteoblast) | NM_001797 | 13.43 |
| FLRT2 | Fibronectin leucine rich transmembrane protein 2 | NM_013231 | 12.03 |
| PCDHB16 | Protocadherin beta 16 | NM_020957 | 11.72 |
| NRCAM | Neuronal cell adhesion molecule, transcript variant 2 | NM_005010 | 10.97 |
| CNTNAP3 | Contactin associated protein-like 3 | NM_033655 | 10.29 |
| GPR98 | G protein-coupled receptor 98, transcript variant 1 | NM_032119 | 10.22 |
| URP2 | UNC-112 related protein 2, transcript variant URP2LF | NM_178443 | 9.94 |
| CX3CL1 | Chemokine (C-X3-C motif) ligand 1 | NM_002996 | 9.49 |
| DCBLD2 | Discoidin, CUB and LCCL domain containing 2 | NM_080927 | 8.66 |
| MYBPH | Myosin binding protein H | NM_004997 | 8.53 |
| VCAN | Versican | NM_004385 | 8.42 |
| CUZD1 | CUB and zona pellucida-like domains 1 | NM_022034 | 7.90 |
| LAMA1 | Laminin, alpha 1 | NM_005559 | 7.88 |
| FLRT3 | Fibronectin leucine rich transmembrane protein 3, transcript variant 2 | NM_198391 | 7.69 |
| CLDN19 | Claudin 19 | NM_148960 | 7.68 |
| ADAM12 | ADAM metallopeptidase domain 12 (meltrin alpha), transcript variant 1 | NM_003474 | 7.41 |
| FAT2 | FAT tumor suppressor homolog 2 (Drosophila) | NM_001447 | 7.00 |
| CX3CR1 | Chemokine (C-X3-C motif) receptor 1 | NM_001337 | 6.59 |
| SLAMF7 | SLAM family member 7 | NM_021181 | 6.47 |
| CYFIP2 | Cytoplasmic FMR1 interacting protein 2, transcript variant 2 | NM_001037332 | 6.38 |
| PCDHA11 | Protocadherin alpha 11, transcript variant 1 | NM_018902 | 6.27 |
| FLJ23834 | Hypothetical protein FLJ23834 | NM_152750 | 6.18 |
| ECM2 | Extracellular matrix protein 2, female organ and adipocyte specific | NM_001393 | 6.08 |
| LAMC3 | Laminin, gamma 3 | NM_006059 | 5.76 |
| SCARF1 | Scavenger receptor class F, member 1, transcript variant 4 | NM_145351 | 5.55 |
| PDPN | Podoplanin, transcript variant 2 | NM_198389 | 5.28 |
| APC | Adenomatosis polyposis coli | NM_000038 | 5.21 |
| CNTN2 | Contactin 2 (axonal) | NM_005076 | 5.19 |
| DST | cDNA: FLJ21489 fis, clone COL05450 | AK025142 | 5.03 |
| AK026826 | cDNA: FLJ23173 fis, clone LNG10019 | AK026826 | 5.03 |
| TGFBI | Transforming growth factor, beta-induced, 68kDa | NM_000358 | 5.02 |
| ICAM5 | Intercellular adhesion molecule 5, telencephalin | NM_003259 | -10.98 |
| RHOB | Ras homolog gene family, member B | NM_004040 | -10.80 |
| PVRL1 | Poliovirus receptor-related 1 (herpesvirus entry mediator C; nectin), transcript variant 3 | NM_203286 | -10.73 |
| ABL1 | v-abl Abelson murine leukemia viral oncogene homolog 1, transcript variant b | NM_007313 | -9.03 |
| ABL2 | v-abl Abelson murine leukemia viral oncogene homolog 2 (arg, Abelson-related gene), transcript variant b | NM_007314 | -8.54 |
| NRXN1 | Neurexin 1, transcript variant alpha | NM_004801 | -8.28 |
| SIGLEC1 | Sialic acid binding Ig-like lectin 1, sialoadhesin | NM_023068 | -7.48 |
| BCL2L11 | BCL2-like 11 (apoptosis facilitator), transcript variant 1 | NM_138621 | -7.08 |
| APOA4 | Apolipoprotein A-IV | NM_000482 | -6.91 |
| NEGR1 | Neuronal growth regulator 1 | NM_173808 | -6.11 |
| MLLT4 | Myeloid/lymphoid or mixed-lineage leukemia (trithorax homolog, Drosophila); translocated to, 4, transcript variant 3 | NM_005936 | -5.12 |
|  | | | |
| **Positive regulation of apoptosis** | | | |
| AK124698 | cDNA FLJ42708 fis, clone BRAMY3007311 | AK124698 | 28.30 |
| IL1B | Interleukin 1, beta | NM_000576 | 22.54 |
| MAL | Mal, T-cell differentiation protein, transcript variant a | NM_002371 | 17.84 |
| NQO1 | NAD(P)H dehydrogenase, quinone 1, transcript variant 1 | NM_000903 | 11.64 |
| BCL11B | B-cell CLL/lymphoma 11B (zinc finger protein), transcript variant 1 | NM_138576 | 10.52 |
| CASP1 | Caspase 1, apoptosis-related cysteine peptidase (interleukin 1, beta, convertase), transcript variant alpha | NM_033292 | 9.30 |
| AHRR | Aryl-hydrocarbon receptor repressor | NM_020731 | 8.88 |
| IGFBP3 | Insulin-like growth factor binding protein 3, transcript variant 1 | NM_001013398 | 8.22 |
| MAP2K6 | Mitogen-activated protein kinase kinase 6 | NM_002758 | 7.44 |
| KIAA0367 | KIAA0367 | NM_015225 | 7.25 |
| PREX1 | Phosphatidylinositol 3,4,5-trisphosphate-dependent RAC exchanger 1 | NM_020820 | 7.25 |
| PLG | Plasminogen | NM_000301 | 6.89 |
| MX1 | Myxovirus (influenza virus) resistance 1, interferon-inducible protein p78 (mouse) | NM_002462 | 6.62 |
| SLAMF7 | SLAM family member 7 | NM_021181 | 6.47 |
| INHBA | Inhibin, beta A (activin A, activin AB alpha polypeptide) | NM_002192 | 6.02 |
| RYR2 | Ryanodine receptor 2 (cardiac) | NM_001035 | 5.96 |
| KALRN | Kalirin, RhoGEF kinase, transcript variant 1 | NM_001024660 | 5.94 |
| PRKCA | Protein kinase C, alpha | NM_002737 | 5.87 |
| CUL3 | Cullin 3 | NM_003590 | 5.30 |
| ADRB2 | Adrenergic, beta-2-, receptor, surface | NM_000024 | 5.26 |
| APC | Adenomatosis polyposis coli | NM_000038 | 5.21 |
| VAV1 | Vav 1 oncogene | NM_005428 | 5.18 |
| CASP8 | Caspase 8, apoptosis-related cysteine peptidase, transcript variant E | NM_033358 | 5.08 |
| AL713762 | mRNA; cDNA DKFZp434K1572 (from clone DKFZp434K1572) | AL713762 | -10.85 |
| JMY | Junction-mediating and regulatory protein | NM_152405 | -10.77 |
| NF1 | Neurofibromin 1 (neurofibromatosis, von Recklinghausen disease, Watson disease), transcript variant 2 | NM_000267 | -9.67 |
| TXNIP | Thioredoxin interacting protein | NM_006472 | -9.50 |
| ABL1 | v-abl Abelson murine leukemia viral oncogene homolog 1, transcript variant b | NM_007313 | -9.03 |
| TIA1 | TIA1 cytotoxic granule-associated RNA binding protein, transcript variant 1 | NM_022037 | -6.59 |
| TNFRSF10B | Tumor necrosis factor receptor superfamily, member 10b, transcript variant 1 | NM_003842 | -5.81 |
| HDAC6 | Histone deacetylase 6, mRNA (cDNA clone IMAGE:4179066), complete cds | BC011498 | -5.71 |
| IHPK2 | Inositol hexaphosphate kinase 2, transcript variant 3 | NM_001005910 | -5.48 |
| HIPK1 | Homeodomain interacting protein kinase 1, transcript variant 2 | NM_152696 | -5.44 |
| RXRA | Retinoid X receptor, alpha | NM_002957 | -5.24 |
|  | | | |
| **Transmembrane Transport** | | | |
| SLC22A15 | Solute carrier family 22 (organic cation transporter), member 15 | NM_018420 | 27.91 |
| SLC24A3 | Solute carrier family 24 (sodium/potassium/calcium exchanger), member 3 | NM_020689 | 24.87 |
| AQP9 | Aquaporin 9 | NM_020980 | 23.50 |
| SCN4A | Sodium channel, voltage-gated, type IV, alpha subunit | NM_000334 | 22.37 |
| SCN11A | Voltage-gated sodium channel alpha subunit, alternate splice variant SCN12A-s | AF150882 | 16.52 |
| SLC22A4 | Solute carrier family 22 (organic cation transporter), member 4 | NM_003059 | 14.45 |
| OCA2 | Oculocutaneous albinism II (pink-eye dilution homolog, mouse) | NM_000275 | 14.34 |
| ABCC11 | ATP-binding cassette, sub-family C (CFTR/MRP), member 11, transcript variant 2 | NM_033151 | 11.99 |
| TRPM6 | Transient receptor potential cation channel, subfamily M, member 6 | NM_017662 | 11.73 |
| SVOP | SV2 related protein homolog (rat) | NM_018711 | 10.24 |
| SLC16A6 | Solute carrier family 16, member 6 (monocarboxylic acid transporter 7) | NM_004694 | 8.98 |
| CATSPER3 | Cation channel, sperm associated 3 | NM_178019 | 7.66 |
| PDPN | Podoplanin, transcript variant 1 | NM_006474 | 7.58 |
| CNGA1 | Cyclic nucleotide gated channel alpha 1 | NM_000087 | 7.35 |
| SFXN5 | Sideroflexin 5 | NM_144579 | 7.06 |
| SLC46A1 | Proton-coupled folate transporter | NM_080669 | 6.91 |
| SCN5A | Sodium channel, voltage-gated, type V, alpha subunit, transcript variant 1 | NM_198056 | 6.53 |
| SLC39A8 | Solute carrier family 39 (zinc transporter), member 8 | NM_022154 | 6.34 |
| TPCN2 | Two pore segment channel 2 | NM_139075 | 6.22 |
| SFXN2 | Sideroflexin 2 | NM_178858 | 6.19 |
| SLC9A9 | Solute carrier family 9 (sodium/hydrogen exchanger), member 9 | NM_173653 | 6.05 |
| RYR2 | Ryanodine receptor 2 (cardiac) | NM_001035 | 5.96 |
| SCN8A | Sodium channel, voltage gated, type VIII, alpha subunit | NM_014191 | 5.90 |
| UCP3 | Uncoupling protein 3 (mitochondrial, proton carrier) (UCP3), nuclear gene encoding mitochondrial protein, transcript variant long | NM_003356 | 5.86 |
| SLC25A18 | Solute carrier family 25 (mitochondrial carrier), member 18 | NM_031481 | 5.76 |
| SLC24A1 | mRNA for KIAA0702 protein, partial cds | AB014602 | 5.75 |
| SLC16A13 | Solute carrier family 16, member 13 (monocarboxylic acid transporter 13) | NM_201566 | 5.60 |
| ATP6AP1L | Hypothetical protein LOC92270 (LOC92270) | NM_001017971 | 5.43 |
| ABCC3 | ATP-binding cassette, sub-family C (CFTR/MRP), member 3 | NM_003786 | 5.35 |
| SLC30A6 | Solute carrier family 30 (zinc transporter), member 6 | NM_017964 | 5.31 |
| ATP6V0D2 | ATPase, H+ transporting, lysosomal 38kDa, V0 subunit d2 | NM_152565 | 5.27 |
| SLC8A1 | Solute carrier family 8 (sodium/calcium exchanger), member 1 | NM_021097 | 5.26 |
| ITPR1 | Inositol 1,4,5-triphosphate receptor, type 1 | NM_002222 | 5.18 |
| SLC23A3 | cDNA FLJ31168 fis, clone KIDNE1000152, moderately similar to Mus musculus yolk sac permease-like molecule 1 (YSPL-1) mRNA | AK055730 | 5.16 |
| AQP7 | Aquaporin 7 | NM_001170 | 5.15 |
| KCNH4 | Potassium voltage-gated channel, subfamily H (eag-related), member 4 | NM_012285 | 5.10 |
| SLC13A3 | Solute carrier family 13 (sodium-dependent dicarboxylate transporter), member 3, transcript variant 2 | NM_001011554 | 5.06 |
